# The causal relationship between diabetes mellitus and meningioma: Insights from the genetic perspective

**DOI:** 10.1016/j.ibneur.2026.10.012

**Published:** 2026-10-17

**Authors:** Qi Sun, Lin Chen, Donghai Ren, Chen Chai

**Affiliations:** a Department of Emergency Medicine, Sir Run Run Shaw Hospital, Zhejiang University School of Medicine, Hangzhou, Zhejiang, 310016, China; b Emergency Center, Zhongnan Hospital of Wuhan University, Hubei Provincial Clinical Research Center for Emergency and Resuscitation, WuHan, China; c Emergency Center, The First People's Hospital of Xiantao, Affiliated Hospital of Hubei University of Science and Technology, XianTao, China

**Keywords:** Diabetes mellitus, Meningioma, Genetic variants, Genome-wide association study, Mendelian randomization

## Abstract

**Background:**

Epidemiological evidence reports the inconsistent association between diabetes mellitus and meningioma risk. We conducted a Mendelian randomization (MR) analysis to evaluate the causality between diabetes-related phenotypes and meningioma.

**Methods:**

Genome-wide association study summary statistics for type 1 diabetes (T1D, N = 590,946), type 2 diabetes (T2D, N = 468,298), hemoglobin A1c (HbA1c, N = 146,806), fasting glucose (FG, N = 129,665), and 2-hour glucose post-oral challenge (2hGlu, N = 57,878) were obtained from the MRC-IEU OpenGWAS and MAGIC consortia. Meningioma data (1835 intracranial cases vs. 377,674 controls; 339 spinal cases vs. 500,009 controls) were derived from FinnGen R12. Genetic variants significantly associated with each exposure were selected as instrumental variables. Inverse-variance weighted regression served as the primary analysis, supplemented by MR-Egger, weighted median, and maximum likelihood methods. Sensitivity analyses evaluated heterogeneity, pleiotropy, outliers, and influential variants.Multiple comparisons were corrected using the Benjamini–Hochberg false discovery rate (FDR) procedure.

**Results:**

Genetically predicted liability to T2D was nominally associated with a lower risk of intracranial meningioma (IVW: OR = 0.07, 95% CI: 0.01–0.97, P = 0.047); however, this association did not remain significant after Benjamini–Hochberg FDR correction (FDR-adjusted P = 0.41). No significant associations were found for T1D (P = 0.334), HbA1c (P = 0.082), FG (P = 0.919), 2hGlu (P = 0.955), or spinal meningioma (all P > 0.05). Sensitivity analyses were reported individually for each exposure–outcome pair. Reverse MR provided no evidence supporting a causal effect of intracranial meningioma on T1D, T2D, or HbA1c.

**Conclusion:**

This MR study provides suggestive, but not definitive, evidence for a potential inverse association between genetically predicted T2D liability and intracranial meningioma risk. After FDR correction, this association did not remain significant, and no significant associations were observed for T1D or other diabetes-related traits.

## Highlights

• T2D genetically reduces intracranial meningioma risk (IVW OR = 0.07).
• No causal links for T1D, HbA1c, FG, or 2hGlu with meningioma.
• Bidirectional MR excludes reverse causation between diabetes and meningioma.
• Sensitivity analyses confirm no pleiotropy or heterogeneity biases.
• First MR study differentiating diabetes subtypes in meningioma etiology.

## 1 Introduction

Meningiomas are the most common primary central nervous system (CNS) tumors, accounting for 40.8% of all primary brain and spinal cord neoplasms, with about 10–12% of cases occurring in the spinal compartment (Wiemels et al., 2010, Ostrom et al., 2023). Although the exact pathogenic mechanisms are not fully understood, known risk factors include exposure to ionizing radiation. Increasing evidence suggests that NF2 germline mutations (neurofibromatosis type 2), hormonal imbalances, obesity, previous breast cancer, and traumatic brain injury are potential contributors (Umansky et al., 2008, Braganza et al., 2012, Carroll et al., 1995, Blankenstein et al., 2000, Degeneffe et al., 2023, Avgerinos et al., 2019, Phillips et al., 2002). Surgical resection, especially Simpson grade I resection (en bloc removal of tumor along with involved dura and bone), remains the gold standard for symptomatic or progressive meningiomas (Nanda et al., 2016, Paldor et al., 2016, Simpson, 1957). Despite advances in surgery (Xiao et al., 2021, Wang et al., 2024, Ivanidze et al., 2025), the 5-year recurrence rate after gross total resection stays at 10–15%, while up to 30% of patients face temporal lobe-related complications, including neurocognitive decline or drug-resistant epilepsy (Bommakanti et al., 2016, Meskal et al., 2016, Hendrix et al., 2017, Apra et al., 2018, Waagemans et al., 2011). Currently, there are no medicinal products approved by the Drug Administration (FDA) or the European Medicines Agency (EMA) that effectively prevent tumor progression or recurrence in this context (Bir et al., 2017, Goldbrunner et al., 2021). This unmet therapeutic need underscores the importance of primary prevention strategies that target modifiable risk factors to reduce the disease burden and its associated complications.

The association between diabetes mellitus (DM) and meningioma risk remains debated. While a Swedish cohort study reported a decreased risk in prediabetic patients (hazard ratio [HR] = 0.45, 95% confidence interval [CI]: 0.29–0.71) (Bernardo et al., 2016), a matched case-control study (2027 cases vs. 20,269 controls) suggested a non-significant inverse association (odds ratio [OR] = 0.89, 95% CI: 0.74–1.07) (Seliger et al., 2017). Conversely, a meta-analysis of eight studies surprisingly showed an increased risk (effect size [ES] = 1.17, 95% CI: 1.02–1.35, *P* = 0.027) (Shi et al., 2021a). These mixed results may be due to confounding factors, such as the potential anti-tumor effects of metformin or detection bias (Coyle et al., 2016).

Mendelian randomization (MR) offers an innovative approach to address confounding in observational studies. By using genetically determined exposure variations (such as diabetes-related single-nucleotide polymorphisms (SNPs) as instrumental variables, MR simulates randomized allocation through meiotic segregation. Since an individual's genotype results from a random combination of parental alleles, genetic variation (such as SNPs) is typically independent of most environmental confounders (Hemani et al., 2018a, Tang et al., 2022). If the core assumptions of instrumental variables (namely correlation, independence, and exclusivity) are satisfied, MR can reduce the influence of reverse causation and confounding (Zhou et al., 2021).

Establishing causal links between diabetes and meningioma remains a methodologically challenging task. To address inconsistent observational evidence, this study employed a two-sample MR framework to examine genetic associations of type 1 diabetes (T1D), type 2 diabetes (T2D), Hemoglobin A1c (HbA1c), fasting glucose (FG), and 2 hr glucose post-oral glucose challenge (2hGlu）with meningioma risk, utilizing genetic instruments to infer causality.

## 2 Materials and methods

### 2.1 Study design

The validity of MR analysis relies on three key instrumental variable (IV) assumptions: relevance assumption, which states that the IVs should be significantly associated with diabetes-related phenotypes (T1D, T2D, HbA1c, FG, and 2hGlu independence assumption, which requires the IVs not to be correlated with any confounding factors of diabetes-related phenotypes or meningioma; and exclusion restriction assumption, which means the IVs should not influence the outcome through pathways other than diabetes-related phenotypes.Fig. 1.

**Fig. 1:**
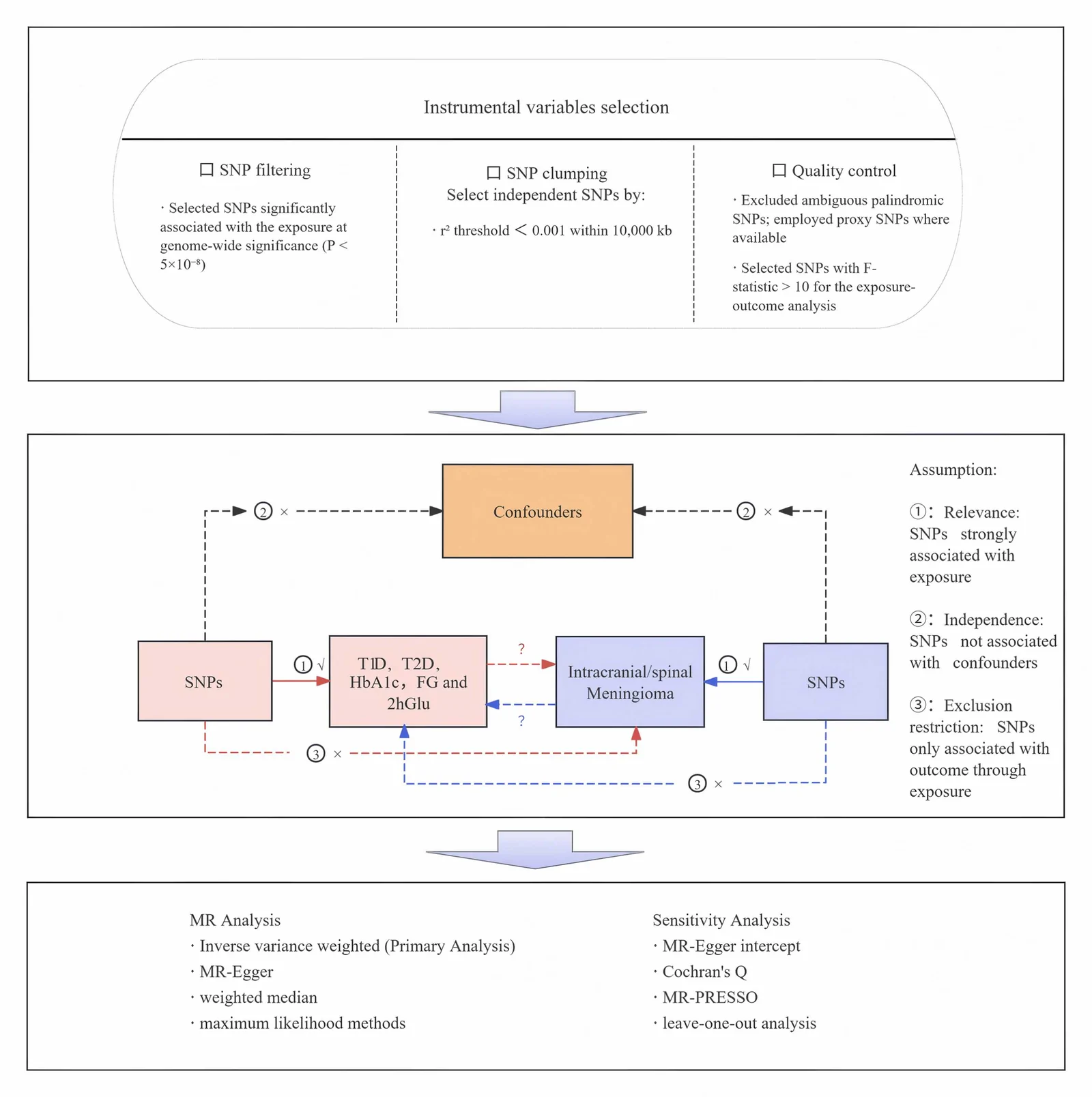
Flowchart illustrating the bidirectional MR study design.

Fig. 1: Flowchart illustrating the bidirectional MR study design. The forward MR examined glycemic traits as exposures and meningioma subtypes (intracranial/spinal) as outcomes, while the reverse MR examined the effects of intracranial meningioma on diabetes-related phenotypes to assess whether there was evidence supporting a causal effect in the reverse direction.

### 2.2 Data sources and ethical approval

The exposure data were derived from summary-level genetic statistics in the MRC Integrative Epidemiology Unit (IEU) OpenGWAS database, including genome-wide association study (GWAS) data for T1D ( dataset ID: ebi-a-GCST90018925; *N* = 590,946 [457,695 Europeans and 133,251 East Asians]), T2D ( ID: ebi-a-GCST90029024; *N* = 468,298 Europeans), and hemoglobin A1c (HbA1c; ID: ebi-a-GCST90002244; *N* = 146,806 Europeans) (Sakaue et al., 2021, Loh et al., 2018, Chen et al., 2021), along with summary statistics for FG (up to 129,665 Europeans) and 2hGlu (57,878 Europeans) from the Meta-Analyses of Glucose and Insulin-related traits Consortium (MAGIC), all of which were used for two-sample MR analysis. Outcome data were derived from the FinnGen Project Database (R12 release), comprising intracranial meningioma (1835 cases versus 377,674 cancer-free controls) and spinal meningioma (339 cases versus 500,009 population controls). Intracranial meningioma was defined using the FinnGen endpoint for brain meningioma excluding all cancers, with case ascertainment based on the cancer register and ICD-O-3 morphology codes. Spinal meningioma was defined using the FinnGen endpoint CD2_BENIGN_MENINGES_SPINAL (Benign neoplasm: Spinal meninges), with case ascertainment based on ICD-10 code D32.1, ICD-9 code 2254, and ICD-8 code 2254, derived from the Finnish Hospital Discharge Registry and Cause of Death Registry. Histological confirmation is not an explicit inclusion criterion in the FinnGen endpoint definitions (Kurki et al., 2023). (Sample sizes for each exposure and outcome GWAS are reported separately in Supplementary Table S1).

### 2.3 The MRC integrative epidemiology unit (IEU) open GWAS database

The MRC IEU Open GWAS database is a publicly accessible resource of GWAS summary statistics created by the University of Bristol research team with funding from the UK MRC, first released in 2020. This comprehensive platform consistently gathers and standardizes publicly available GWAS data from major initiatives, including UK Biobank, FinnGen, and the GWAS Catalog, covering 14,582 distinct studies with around 126 billion genetic associations across various human phenotypes (such as metabolic disorders and cardiovascular traits) and multi-ethnic groups.

### 2.4 The meta-analyses of glucose and insulin-related traits consortium (MAGIC)

MAGIC is an international collaborative research initiative launched in 2008 to explore the genetic basis of glycemic traits through large-scale meta-analyses. MAGIC combines GWAS data from numerous cohorts around the world, covering key metabolic parameters such as FG, 2hGlu, fasting insulin, and insulin resistance.

The MAGIC consortium includes diverse ancestral groups (European, African, East Asian, and South Asian populations) to improve the broad applicability of genetic findings.

### 2.5 FinnGen project database

FinnGen is a nationwide genomic research initiative based on Finnish biobanks, launched in 2017 to promote precision medicine by combining genomic data with electronic health records (EHRs). As of the R12 release (2023), the project has enrolled over 500,000 participants, covering a wide range of disease phenotypes including cancer, cardiovascular disorders, neurological diseases, and metabolic conditions.

The FinnGen study received ethical approval from the Finnish Ethics Committee (Approval No. HUS/990/2017), and all participants provided written informed consent. Data use complies with the European Union's General Data Protection Regulation (GDPR).

All studies involved received approval from local ethics committees and followed the principles of the Declaration of Helsinki, with written informed consent from all participants.

### 2.6 Instrumental variable selection

We systematically identified SNPs significantly associated with diabetes-related phenotypes (T1D, T2D, HbA1c, FG, and 2hGlu) based on GWAS summary statistics. SNPs significantly associated with T1D, T2D, HbA1c, FG, and 2hGlu were selected based on a threshold of *P* < 5.0 × 10⁻⁸ to ensure robust association with exposure. Subsequently, threshold parameters were set (r² < 0.001 within 10,000 kb) to minimize linkage disequilibrium effects (Myers et al., 2020). The F-statistic for each instrumental variable was calculated to reduce weak instrument bias, with values above 10 indicating strong instruments. When harmonizing exposure and outcome data, palindromic SNPs that could cause strand ambiguity were excluded. For missing variants, we identified and utilized proxy SNPs when available. The final set of quality-controlled SNPs was then used for causal inference analyses. Additionally, we rigorously adhered to the STROBE-MR reporting guidelines to ensure methodological transparency and completeness (Little et al., 2009, Richardson et al., 2020). This study included validated and independent genetic variants associated with diabetes-related phenotypes as instrumental variables. For each exposure–outcome analysis, we report the number of SNPs initially selected as instrumental variables, the number removed during harmonization, and the final number included in the MR analysis. The discrepancy between the number of selected instruments and the degrees of freedom in heterogeneity tests reflects SNP removal during harmonization and the exclusion of palindromic or ambiguous SNPs. (see Supplementary Table S2 online).

### 2.7 Bidirectional two-sample MR analyses

The two-sample MR was employed to evaluate the potential causal associations between diabetes-related phenotypes (T1D, T2D, HbA1c, FG, and 2hGlu) and meningioma (including intracranial meningioma and spinal meningioma). All analyses adhered to the STROBE-MR reporting guidelines for genetic causal inference studies. The IVW method served as the primary analytical approach, as it provides unbiased estimates under the assumption that all IVs are valid (i.e., no horizontal pleiotropy). To ensure robustness, we supplemented IVW with three additional methods: the weighted median method, MR-Egger regression, and maximum likelihood analysis (Burgess et al., 2019).

To account for multiple comparisons, we applied the Benjamini–Hochberg false discovery rate (FDR) procedure using the p.adjust function in R (method = "BH") across the 10 primary exposure–outcome comparisons (5 diabetes-related exposures × 2 meningioma subtypes). Reverse MR analyses were corrected separately. Associations with FDR-adjusted P < 0.05 were considered statistically significant; associations that were significant only before correction were described as nominal or suggestive.

For binary exposures such as T2D, the MR estimate represents the effect of genetically predicted liability to T2D rather than the direct effect of clinical T2D status. The OR should therefore be interpreted on the scale of genetic liability to T2D, not as the effect of a clinical diagnosis of T2D.

Reverse MR analyses were performed using intracranial meningioma as the exposure and T1D, T2D, HbA1c, FG, and 2hGlu as outcomes. These analyses should be interpreted with caution because they relied on a limited number of genetic instruments or single-SNP Wald ratio estimates and therefore had limited statistical power. Reverse MR analyses were not performed for spinal meningioma because of insufficient valid genetic instruments.

### 2.8 Sensitivity analyses

The MR analysis used Cochran’s Q test to evaluate heterogeneity among IVs. If significant heterogeneity was detected (*P* ≤ 0.05), random-effects IVW was applied; otherwise, fixed-effects IVW was applied (Xiao et al., 2022). Horizontal pleiotropy was evaluated via the MR-Egger intercept test, with a non-significant intercept (*P* > 0.05) indicating no detectable pleiotropy (Bowden et al., 2015). Potential outliers were identified and removed iteratively using MR-PRESSO (*P* < 0.05), and causal estimates were recalculated after each exclusion (Verbanck et al., 2018). Additionally, leave-one-out sensitivity analysis was performed by sequentially removing a single SNP and recalculating the combined effect size of the remaining SNPs to assess the impact of each SNP on the causal relationship.

All statistical analyses were conducted in R (v 4.3.3) using the TwoSampleMR package (v0.5.5) (Hemani et al., 2018b) and the MRPRESSO package (v1.0).

## 3 Results

### 3.1 Causal effects of T2D on intracranial meningioma

We implemented a rigorous, multi-stage quality control process to select valid genetic instruments that represent diabetes-related exposure phenotypes. The final set of SNPs meeting all predefined inclusion criteria (genome-wide significance threshold: *P* < 5 ×10⁸; instrument strength: F-statistic > 10) was retained, with all detailed information provided in Supplementary Table S2. This process yielded 67 independent SNPs as instruments for T2D (see Supplementary Table S2).

Forward MR showed a nominally significant inverse association between genetically predicted T2D and intracranial meningioma risk using the IVW method (OR = 0.07, 95% CI: 0.01–0.97, P = 0.047). However, after Benjamini–Hochberg FDR correction for the 10 primary exposure–outcome comparisons, this association did not remain statistically significant (FDR-adjusted P = 0.41). Therefore, this finding should be interpreted as a nominal or suggestive association rather than definitive evidence of causality.Fig. 2 shows a forest plot illustrating the estimated effects of individual T2D-associated SNPs on intracranial meningioma risk. Out of the 67 selected T2D-associated SNPs, five variants (rs7274168, rs76895963, rs6446298, rs4287436, and rs11257655) showed statistically significant individual effects on meningioma risk, whereas the remaining SNPs (e.g., rs2510078, rs76665052) did not.

**Fig. 2:**
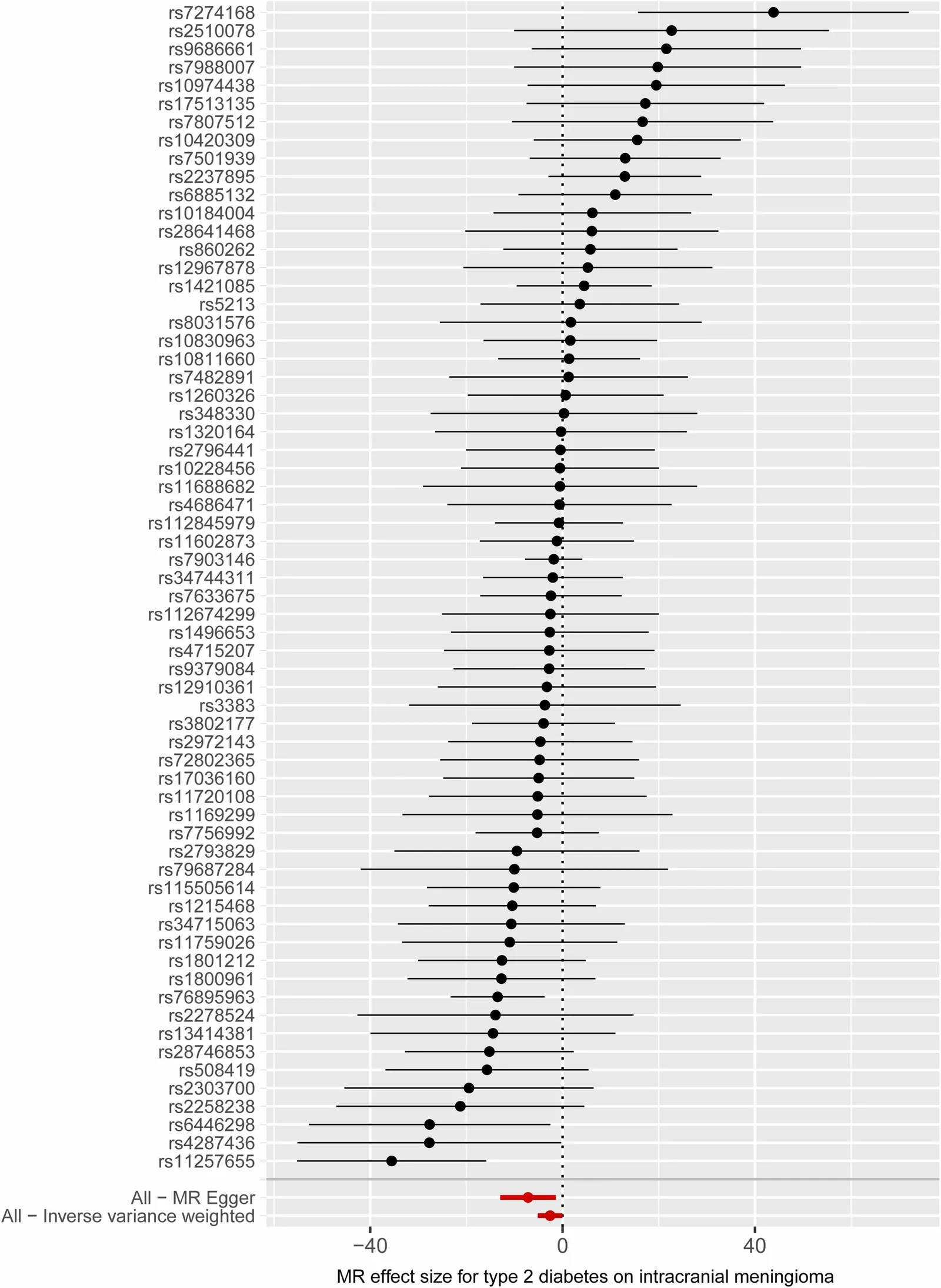
Mendelian randomization estimates for the causal effect of T2D and intracranial meningioma.

In sensitivity analyses, MR-Egger (P = 0.018) and maximum likelihood estimation (OR = 0.07, 95% CI: 0.00–0.74, P = 0.027) showed results in the same direction, with no evidence of directional pleiotropy (MR-Egger intercept P = 0.090). In contrast, the weighted median estimate was not significant (OR = 0.14, 95% CI: 0.003–7.13, P = 0.329). Reverse MR analysis showed no association between genetically predicted meningioma and T2D risk (IVW P = 0.241) (Table 1). Although the inverse association was directionally consistent across several analytical approaches and leave-one-out analysis did not identify any single influential variant, the wide confidence interval and the non-significant FDR-adjusted P value indicate that this finding should be interpreted with caution as suggestive rather than conclusive.

**Table 1:** Two-sample MR estimates for the causal effect of T1D, T2D, HbA1c, FG, and 2hG on intracranial meningioma.

Exposure	Outcome	Method	Beta	Standard error	Pvalue
Type 1 diabetes	Intracranial meningioma	Inverse variance weighted	0.033	0.034	0.334
		MR Egger	-0.072	0.051	0.182
		Maximum likelihood	0.033	0.031	0.293
		Weighted median	0.000	0.048	0.999
Type 2 diabetes	Intracranial meningioma	Inverse variance weighted	-2.594	1.306	0.047
		MR Egger	-7.182	2.958	0.018
		Maximum likelihood	-2.638	1.192	0.027
		Weighted median	-1.946	1.995	0.329
Hemoglobin levels	Intracranial meningioma	Inverse variance weighted	-0.625	0.359	0.082
		MR Egger	-0.504	0.699	0.473
		Maximum likelihood	-0.628	0.306	0.040
		Weighted median	-0.050	0.499	0.921
Fasting glucose	Intracranial meningioma	Inverse variance weighted	0.022	0.221	0.919
		MR Egger	-0.410	0.404	0.317
		Maximum likelihood	0.023	0.222	0.918
		Weighted median	0.076	0.317	0.811
2 hr glucose post-oral glucose challenge	Intracranial meningioma	Inverse variance weighted	0.009	0.157	0.955
		MR Egger	0.138	0.553	0.807
		Maximum likelihood	0.009	0.155	0.952
		Weighted median	-0.033	0.212	0.875

### 3.2 Null associations of T1D，HbA1c, FG, and 2hGlu with meningioma subtypes

Bidirectional MR analyses showed no statistically significant associations between T1D and either intracranial or spinal meningioma (IVW P = 0.334 and P = 0.648, respectively), nor between HbA1c (intracranial: IVW P = 0.082; spinal: IVW P = 0.410), FG (intracranial: IVW P = 0.919; spinal: IVW P = 0.930), or 2hGlu (intracranial: IVW P = 0.955; spinal: IVW P = 0.646) and meningioma risk. After Benjamini–Hochberg FDR correction for the 10 primary exposure–outcome comparisons, none of these associations remained significant (all FDR-adjusted P ≥ 0.41). Similarly, reverse MR analyses provided no evidence that intracranial meningioma affects T1D, T2D, HbA1c, FG, or 2hGlu (all P > 0.05).

Sensitivity analyses were not uniform across all exposure–outcome pairs and are therefore reported individually. Evidence of directional pleiotropy was observed in the T1D–intracranial meningioma analysis (MR-Egger intercept P = 0.020); as pleiotropy may bias the estimate toward the null, this null finding should be interpreted with caution. Significant heterogeneity was detected for HbA1c–intracranial meningioma (Cochran's Q P = 0.016), and random-effects IVW was applied. For this pair, the IVW estimate was not significant (P = 0.082), whereas the maximum likelihood estimate was nominally significant (P = 0.016), indicating that the result is inconclusive rather than definitively null. No evidence of directional pleiotropy or heterogeneity was observed for the remaining exposure–outcome pairs (MR-Egger intercept P > 0.05; Cochran's Q P > 0.05).

### 3.3 Sensitivity analysis

Sensitivity analyses were performed for each exposure–outcome pair and are reported individually. For the primary T2D–intracranial meningioma analysis, no evidence of directional pleiotropy (MR-Egger intercept P = 0.090) or significant heterogeneity (Cochran's Q P = 0.111) was observed. Leave-one-out analysis did not identify any single influential variant, and the funnel plot was symmetric (Fig. 3, Fig. 4).

**Fig. 3:**
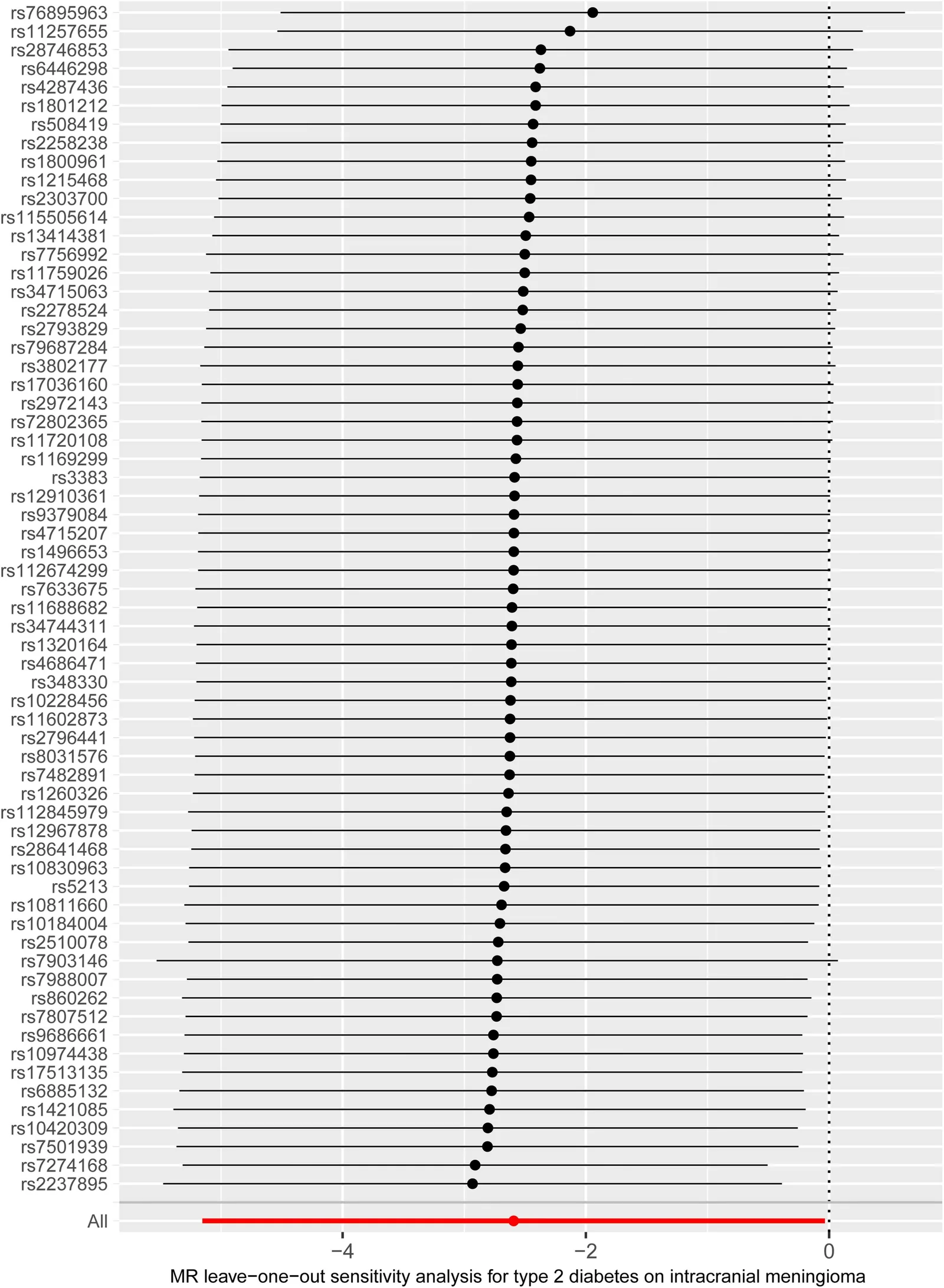
Leave-one-out analysis of T2D and intracranial meningioma.

**Fig. 4:**
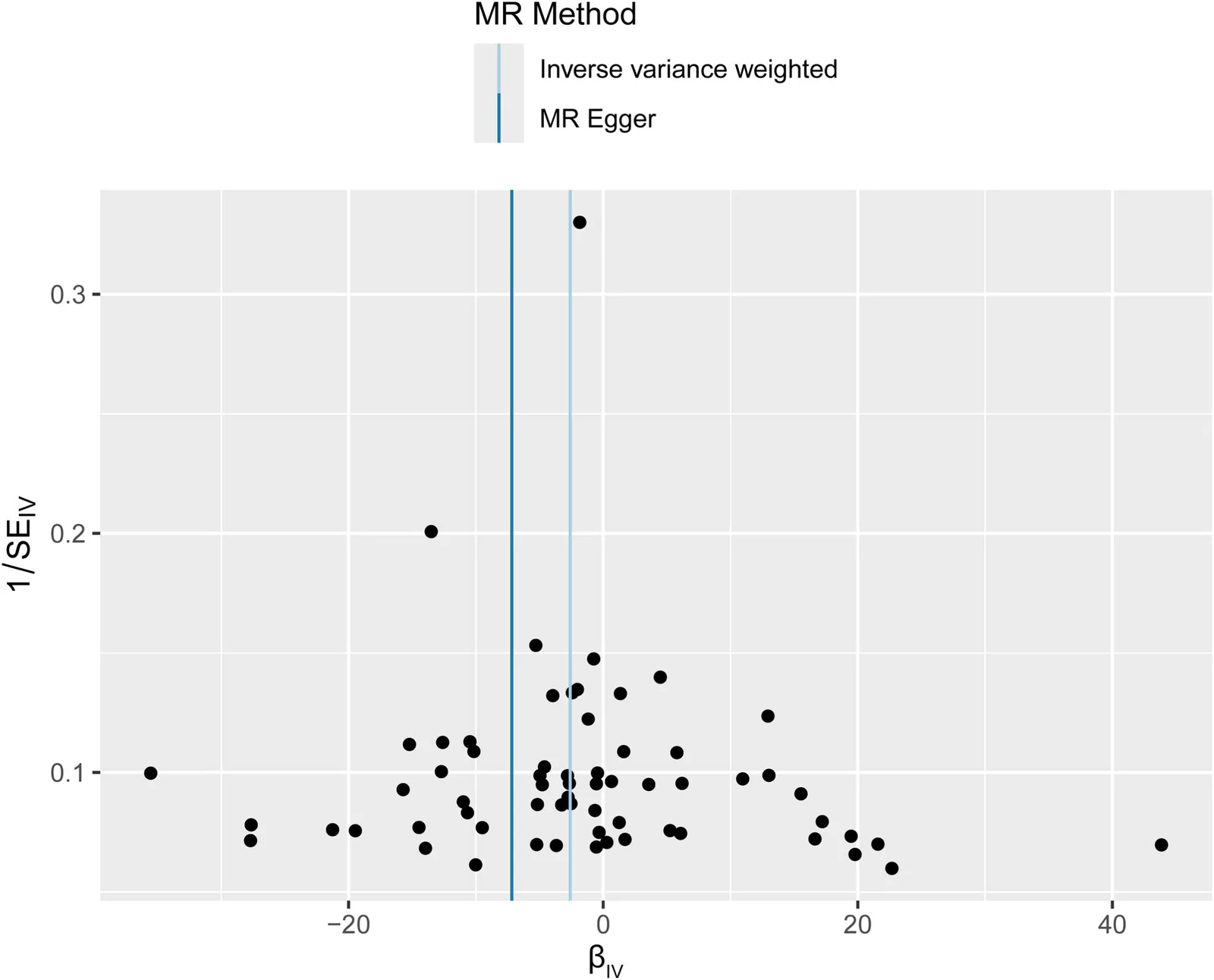
The funnel plots of the heterogeneity test of mendelian randomization analysis in intracranial meningiom.

For the remaining exposure–outcome pairs, sensitivity results were not uniform. Directional pleiotropy was observed for T1D–intracranial meningioma (MR-Egger intercept P = 0.020), and significant heterogeneity was detected for HbA1c–intracranial meningioma (Cochran's Q P = 0.016), for which random-effects IVW was applied. No evidence of pleiotropy or heterogeneity was observed for the remaining pairs (MR-Egger intercept P > 0.05; Cochran's Q P > 0.05)(Detailed sensitivity results are provided in Supplementary Table S3).

## 4 Discussion

The relationship between DM and meningiomas remains unclear and controversial due to inconsistent epidemiological findings. Because intracranial and spinal meningiomas may differ in their biological characteristics and clinical behavior, we analyzed these two outcomes separately rather than combining them. To address these inconsistencies,we systematically examined the genetic basis of the causal link between diabetes-related phenotypes and meningiomas. We used genome-wide association analysis and identified key SNPs with independent inheritance as IVs to evaluate causality between exposure and outcome. Genetic instrumental methods provided empirical evidence of causality, reducing potential confounding or reverse causation issues. Our results showed a nominally significant negative association between genetically predicted T2D and intracranial meningioma; however, this association did not remain significant after Benjamini–Hochberg FDR correction for multiple comparisons. No significant associations were found between other diabetes-related phenotypes (T1D, HbA1c, FG, and 2hGlu) and meningioma. In addition, reverse MR analyses provided no evidence supporting a causal effect of meningioma on diabetes-related phenotypes, although these analyses had limited statistical power because some relied on few genetic instruments.The magnitude of the OR should be interpreted with caution, as it reflects the effect of genetically predicted liability to T2D rather than the direct effect of clinical T2D status. It does not imply that clinical T2D itself directly reduces meningioma risk.

Previous observational studies have yielded conflicting evidence. While A German case-control study (*N* = 306) found that pre-existing diabetes was significantly associated with meningioma in middle-aged patients (40–69 years) (Schneider et al., 2005), a systematic review and meta-analysis indicated that diabetes increases the risk of meningioma in both women and men (Shi et al., 2021b). Conversely, the Swedish AMORIS cohort study (*N* = 296) found an inverse relationship between diabetes and overall meningioma risk (HR = 0.45, 95% CI: 0.29–0.71), with metformin use proposed as a potential explanation (Bernardo et al., 2016). Notably, a matched case-control study involving 2027 meningioma cases and 20,269 controls demonstrated that diabetes was inversely associated with meningioma (OR = 0.89; 95% CI = 0.74–1.07), mainly driven by an inverse association among women (OR = 0.78; 95% CI = 0.62–0.98). There is no significant correlation between HbA1c levels and meningioma risk. Unexpectedly, a significant positive association with metformin was revealed after adjustment for diabetes duration and HbA1c levels (Seliger et al., 2017). An earlier cohort study found no significant relationship between FG and meningioma risk (Edlinger et al., 2012). However, another population-based international case-control study conducted across eight centers in six countries revealed an inverse association between FG and meningioma risk in women (Schlehofer et al., 1999). These conflicting results underscore the urgent need to clarify this relationship using genetic causal inference methods.

Our study provides suggestive evidence for an inverse relationship between genetically predicted T2D liability and intracranial meningioma risk, as shown through Mendelian randomization analysis. Notably, this inverse relationship is specific to T2D, with no causal effects observed for T1D or other diabetes-related traits (HbA1c, FG, and 2hGlu). This distinction could suggest that the reduced meningioma risk might be driven by unique metabolic changes in T2D, such as chronic hyperinsulinemia and insulin resistance, rather than hyperglycemia itself, although mechanistic studies are required to confirm this hypothesis.

While HbA1c, FG, and 2hGlu are well-established markers of short- to medium-term glycemic control, they primarily reflect glucose homeostasis rather than the broader metabolic dysregulation in T2D. Chronic hyperinsulinemia, insulin resistance, and altered adipokine signaling—hallmarks of T2D—could potentially influence meningioma development through their effects on cellular growth (such as via IGF-1 pathways) and inflammation. In contrast, acute glucose swings (measured by FG/2hGlu) or overall glycemic exposure (HbA1c) likely have a minimal impact, which explains the lack of observed associations.

Several potential biological mechanisms could support our findings. The inverse association between T2D and meningioma risk may involve changes in sex hormone pathways, especially given the established presence of progesterone and androgen receptors in meningiomas (Wiemels et al., 2010, Blankenstein et al., 2000, Chen and Chen, 2001),and the suggestion that female sex hormones increase the risk of meningioma (Benson et al., 2015, Claus et al., 2013). This idea is supported by studies showing that women with diabetes experience a partial decline in ovarian ability to convert androgens into estrogens (Stamataki et al., 1996), and the inverse association between diabetes and meningioma risk is not seen in women who receive exogenous estrogen supplementation (Seliger et al., 2017, Wigertz et al., 2006). At the same time, in T2D, insulin resistance causes characteristic endocrine changes, where hyperinsulinemia reduces hepatic sex hormone-binding globulin (SHBG) production, resulting in higher levels of circulating free testosterone and estradiol. Although meningiomas have hormone receptors that could respond to these changes, the overall effect appears to be protective. Experimental results suggest that increased free testosterone may have anti-tumor effects through activation of the androgen receptor (Wittert and Grossmann, 2022). This complex hormonal interaction probably creates an environment where growth-inhibiting signals outweigh any potential proliferative stimuli. Like other tumors, meningiomas require large amounts of glucose to build cell walls and support cell proliferation (Bharadwaj et al., 2015, Vander Heiden et al., 2009). This process is known as the Warburg effect, and β-cell failure caused by long-term T2D may lead to a hypoinsulinemic state that decreases glucose uptake by tumor cells, thereby slowing their growth (Bharadwaj et al., 2015). On the other hand, meningioma growth depends on angiogenesis, which requires the formation of new blood vessels to support tumor development (Preusser et al., 2012). Chronic hyperglycemia in diabetes causes microvascular endothelial dysfunction through advanced glycation end product (AGE) buildup, oxidative stress, and impaired Vascular Endothelial Growth Factor (VEGF) signaling. These pathological changes may compromise tumor neovascularization, creating an unfavorable environment for meningioma growth (Chung et al., 2015).T1D is now considered an autoimmune disease involving genetic, immune, and environmental factors (Acharjee et al., 2013), while T2D affects multiple tissues through chronic, systemic low-grade inflammation rather than autoimmune responses (Giovannucci et al., 2010, Velloso et al., 2013).it has been found that patients with T1D have a reduced number of circulating neutrophils, which may be related to an autoimmune response. In contrast, patients with T2D show a more significant increase in circulating inflammatory proteins (Obeagu and Obeagu, 2023, van Heck et al., 2025). This variation in inflammatory response partly explains the differences between the two types of diabetes mellitus in the incidence and severity of tumor-related complications (Jende et al., 2018). Further research is needed to explore the relationship between meningioma risk and different types of diabetes.

Several strengths of our study are worth highlighting. To our knowledge, this is among the first MR studies to examine the potential association between diabetes-related traits and meningioma risk, and our analysis reduced the influence of residual confounding and reverse causation inherent in observational studies through carefully selected genetic instruments. We used the IVW method as our primary approach, supported by sensitivity analyses including MR-Egger regression, weighted median estimator, and MR-PRESSO. Notably, this study conducted separate analyses for diabetes-related phenotypes (T1D, T2D, HbA1c, FG, and 2hGlu), providing new evidence about their different associations with meningioma risk. Despite these strengths, several limitations should be considered when interpreting our results. First, the genetic instruments were mainly derived from GWAS data of European ancestry, which may limit the applicability of our findings to other ethnic populations. Second, based on strong epidemiological evidence showing a significantly higher incidence of meningioma in women compared to men, the potential sex-specific effects of our observed associations need further exploration in stratified analyses. The currently available GWAS data, due to gender-agnostic data, makes meaningful gender subgroup analysis methodologically challenging. Third, while Mendelian randomization minimizes confounding by post-diagnosis factors, we cannot completely exclude potential influences of glucose-lowering medications (e.g., metformin's proposed anti-tumor effects), warranting investigation in pharmacoepidemiologic studies with detailed treatment data. Fourth, while our Mendelian randomization analysis identifies genetic associations, the functional characterization of implicated SNPs in meningeal tissue requires future epigenomic and mechanistic studies. In particular, investigation of chromatin accessibility, DNA methylation patterns, and histone modifications in meningiomas from diabetic versus non-diabetic patients may help elucidate the observed inverse association. Finally, the inverse association between genetically predicted T2D liability and intracranial meningioma was only nominally significant and did not remain significant after Benjamini–Hochberg FDR correction for multiple comparisons. Given the limited number of intracranial meningioma cases (1835 cases) and the wide confidence interval (0.01–0.97), this finding should be interpreted as suggestive rather than definitive, and replication in larger meningioma GWAS datasets will be needed to confirm it.

## 5 Conclusion

This MR study provides suggestive, but not definitive, evidence for a potential inverse association between genetically predicted T2D liability and intracranial meningioma risk. After Benjamini–Hochberg FDR correction for multiple comparisons, this association did not remain statistically significant, and no significant associations were observed for T1D or other diabetes-related traits. Further studies are needed to confirm these findings and clarify the underlying mechanism.

## CRediT authorship contribution statement

**Qi Sun:** Writing – original draft, Software, Resources, Methodology, Formal analysis, Data curation, Conceptualization. **Lin Chen:** Writing – original draft, Methodology, Formal analysis, Data curation, Conceptualization, Writing – review & editing, Writing – original draft, Visualization, Validation, Supervision, Software, Resources, Project administration, Methodology, Investigation, Funding acquisition, Formal analysis, Data curation, Conceptualization. **Donghai Ren:** Writing – original draft, Methodology, Formal analysis, Data curation, Conceptualization. **Chen Chai:** Writing – review & editing, Writing – original draft, Visualization, Validation, Supervision, Software, Resources, Project administration, Methodology, Investigation, Funding acquisition, Formal analysis, Data curation, Conceptualization.

## Ethical approval

This study exclusively used publicly available summary-level data from previously published genome-wide association studies. All original studies received approval from their respective institutional review boards and ethics committees, and all participants provided written informed consent. As this was a secondary analysis of de-identified, publicly accessible data, no additional ethical approval or informed consent was required.

## Ethics declaration

This study did not require additional informed consent, as it exclusively used publicly accessible data. All research procedures had previously obtained approval from the relevant institutional review boards.

## Consent for publication

Not applicable.

## Funding

This study was supported by the China Medical Foundation (Grant Number: 2025CMFC5). The funder had no role in study design, data collection, analysis, decision to publish, or preparation of the manuscript.

## Conflict of Interest

The authors declare no competing financial or non-financial interests.

## Data Availability

The datasets analyzed in this study are publicly available. Summary statistics for type 1 diabetes, type 2 diabetes, and hemoglobin A1c were obtained from the MRC-IEU OpenGWAS database (https://gwas.mrcieu.ac.uk/). Fasting glucose and 2-hour glucose post-oral challenge data were obtained from the MAGIC consortium. Meningioma outcome data were derived from the FinnGen project R12 release (https://www.finngen.fi/). All data sources are cited in the references.

## Footnotes

Appendix A Supplementary data associated with this article can be found in the online version at doi:10.1016/j.ibneur.2026.10.012.
