# Supplementary material for: The causal relationship between diabetes mellitus and meningioma: Insights from the genetic perspective
Source: IBRO Neurosci Rep. 2026 Oct 17;21:1077–86. doi: 10.1016/j.ibneur.2026.10.012 (PMC13743375; doi:10.1016/j.ibneur.2026.10.012)
Supplement: Supplementary file 1 [file mmc1.docx]

**Supplementary Materials**

**Supplementary Table S1.** Information of genetic association study summary data.

**Supplementary Table S2.** Association of T1D, T2D, HbA1c, FG, and 2hGlu genetic instrumental variants with meningioma GWAS.

**Supplementary Table S3.** MR estimates for the causal effect of diabetes and meningioma.

**Supplementary Table S4.** MR estimates and sensitivity analyses for the causal associations between diabetes-related traits and meningioma, with Benjamini–Hochberg FDR-adjusted P values.

**Supplementary Table S1.** Information of genetic association study summary data.

| type 1 diabetes  ebi-a-GCST90018925 | Sakaue et al. | 590,946 samples | European and East Asians | Sakaue, S., et al., A cross-population atlas of genetic associations for 220 human phenotypes. Nat Genet, 2021. 53(10): p. 1415-1424. |
| --- | --- | --- | --- | --- |
| type 2 diabetes  ebi-a-GCST90029024 | Loh et al. | 468,298 samples | European | Loh, P.R., et al., Mixed-model association for biobank-scale datasets. Nat Genet, 2018. 50(7): p. 906-908. |
| Hemoglobin A1c  ebi-a-GCST90002244 | Chen et al. | 146,806 samples | European | Chen, J., et al., The trans-ancestral genomic architecture of glycemic traits. Nat Genet, 2021. 53(6): p. 840-860. |
| fasting glucose  https://magicinvestigators.org/downloads/index.html | Willems et al. | 129,665 samples | European | Willems SM., et al., Large-scale exome array summary statistics resources for glycemic traits to aid effector gene prioritization. Wellcome Open Res. 2023 Oct 20;8:483. |
| 2hr glucose post-oral glucose  https://magicinvestigators.org/downloads/index.html | Willems et al. | 57,878 samples | European | Willems SM., et al., Large-scale exome array summary statistics resources for glycemic traits to aid effector gene prioritization. Wellcome Open Res. 2023 Oct 20;8:483. |
| intracranial meningioma  https://r12.finngen.fi/pheno/C3_MENINGIOMA_EXALLC | Kurki et al. | 379,509 samples | Finland | Kurki, M.I., et al., FinnGen provides genetic insights from a well-phenotyped isolated population. Nature, 2023. 613(7944): p. 508-518. |
| spinal meningioma  https://r12.finngen.fi/pheno/CD2_BENIGN_MENINGES_SPINAL | Kurki et al. | 500,348 samples | Finland | Kurki, M.I., et al., FinnGen provides genetic insights from a well-phenotyped isolated population. Nature, 2023. 613(7944): p. 508-518. |

**Supplementary Table S2.** Association of T1D, T2D, HbA1c, FG, and 2hGlu genetic instrumental variants with meningioma GWAS.

| SNP | exposure | beta.exposure | se.exposure | pval.exposure | outcome | beta.outcome | se.outcome | pval.outcome | R^2^ | F-value |
| --- | --- | --- | --- | --- | --- | --- | --- | --- | --- | --- |
| rs10811662 | Type 1 diabetes | -0.141 | 0.022 | 1.14e-10 | Intracranial meningioma | -0.007 | 0.048 | 0.878 | 0.000 | 41.570 |
| rs10876866 | Type 1 diabetes | 0.110 | 0.020 | 2.69e-08 | Intracranial meningioma | 0.037 | 0.036 | 0.305 | 0.000 | 30.833 |
| rs115380430 | Type 1 diabetes | 0.837 | 0.089 | 3.45e-21 | Intracranial meningioma | -0.021 | 0.108 | 0.845 | 0.000 | 89.266 |
| rs13204736 | Type 1 diabetes | 0.516 | 0.022 | 3.30e-123 | Intracranial meningioma | 0.040 | 0.034 | 0.229 | 0.001 | 559.602 |
| rs17711850 | Type 1 diabetes | 0.124 | 0.021 | 1.93e-09 | Intracranial meningioma | 0.055 | 0.038 | 0.156 | 0.000 | 36.000 |
| rs189853531 | Type 1 diabetes | 0.687 | 0.118 | 5.81e-09 | Intracranial meningioma | -0.034 | 0.212 | 0.871 | 0.000 | 33.924 |
| rs192460891 | Type 1 diabetes | -0.286 | 0.024 | 2.38e-32 | Intracranial meningioma | -0.093 | 0.043 | 0.031 | 0.000 | 140.437 |
| rs2847278 | Type 1 diabetes | -0.155 | 0.026 | 1.42e-09 | Intracranial meningioma | -0.073 | 0.047 | 0.123 | 0.000 | 36.562 |
| rs28891414 | Type 1 diabetes | -0.305 | 0.023 | 5.24e-41 | Intracranial meningioma | -0.021 | 0.047 | 0.662 | 0.000 | 180.174 |
| rs3184504 | Type 1 diabetes | -0.143 | 0.019 | 3.76e-14 | Intracranial meningioma | -0.007 | 0.034 | 0.837 | 0.000 | 57.487 |
| rs3842753 | Type 1 diabetes | 0.308 | 0.022 | 8.31e-44 | Intracranial meningioma | -0.030 | 0.041 | 0.464 | 0.000 | 193.109 |
| rs6679677 | Type 1 diabetes | 0.426 | 0.029 | 8.96e-50 | Intracranial meningioma | 0.049 | 0.047 | 0.295 | 0.000 | 219.306 |
| rs706779 | Type 1 diabetes | -0.096 | 0.017 | 3.23e-08 | Intracranial meningioma | -0.010 | 0.034 | 0.773 | 0.000 | 30.630 |
| rs7167984 | Type 1 diabetes | -0.126 | 0.023 | 2.75e-08 | Intracranial meningioma | -0.017 | 0.046 | 0.711 | 0.000 | 30.908 |
| rs72805613 | Type 1 diabetes | 0.098 | 0.018 | 4.04e-08 | Intracranial meningioma | 0.035 | 0.034 | 0.307 | 0.000 | 30.096 |
| rs7567242 | Type 1 diabetes | 0.142 | 0.024 | 4.17e-09 | Intracranial meningioma | 0.095 | 0.048 | 0.047 | 0.000 | 34.522 |
| rs7903146 | Type 1 diabetes | 0.167 | 0.022 | 1.48e-14 | Intracranial meningioma | -0.025 | 0.042 | 0.543 | 0.000 | 59.084 |
| rs9273363 | Type 1 diabetes | 0.730 | 0.020 | 1.00e-200 | Intracranial meningioma | -0.051 | 0.037 | 0.171 | 0.003 | 1,402.977 |
| rs10811662 | Type 1 diabetes | -0.141 | 0.022 | 1.14e-10 | Spinal meningioma | -0.025 | 0.109 | 0.818 | 0.000 | 41.570 |
| rs10876866 | Type 1 diabetes | 0.110 | 0.020 | 2.69e-08 | Spinal meningioma | 0.123 | 0.082 | 0.134 | 0.000 | 30.833 |
| rs115380430 | Type 1 diabetes | 0.837 | 0.089 | 3.45e-21 | Spinal meningioma | -0.122 | 0.255 | 0.633 | 0.000 | 89.266 |
| rs13204736 | Type 1 diabetes | 0.516 | 0.022 | 3.30e-123 | Spinal meningioma | 0.035 | 0.077 | 0.646 | 0.001 | 559.602 |
| rs17711850 | Type 1 diabetes | 0.124 | 0.021 | 1.93e-09 | Spinal meningioma | -0.091 | 0.089 | 0.305 | 0.000 | 36.000 |
| rs189853531 | Type 1 diabetes | 0.687 | 0.118 | 5.81e-09 | Spinal meningioma | 0.137 | 0.481 | 0.777 | 0.000 | 33.924 |
| rs192460891 | Type 1 diabetes | -0.286 | 0.024 | 2.38e-32 | Spinal meningioma | -0.011 | 0.099 | 0.91 | 0.000 | 140.437 |
| rs2847278 | Type 1 diabetes | -0.155 | 0.026 | 1.42e-09 | Spinal meningioma | -0.065 | 0.110 | 0.55 | 0.000 | 36.562 |
| rs28891414 | Type 1 diabetes | -0.305 | 0.023 | 5.24e-41 | Spinal meningioma | 0.003 | 0.110 | 0.975 | 0.000 | 180.174 |
| rs3184504 | Type 1 diabetes | -0.143 | 0.019 | 3.76e-14 | Spinal meningioma | -0.025 | 0.078 | 0.747 | 0.000 | 57.487 |
| rs3842753 | Type 1 diabetes | 0.308 | 0.022 | 8.31e-44 | Spinal meningioma | -0.012 | 0.095 | 0.898 | 0.000 | 193.109 |
| rs6679677 | Type 1 diabetes | 0.426 | 0.029 | 8.96e-50 | Spinal meningioma | 0.069 | 0.109 | 0.528 | 0.000 | 219.306 |
| rs706779 | Type 1 diabetes | -0.096 | 0.017 | 3.23e-08 | Spinal meningioma | -0.071 | 0.079 | 0.371 | 0.000 | 30.630 |
| rs7167984 | Type 1 diabetes | -0.126 | 0.023 | 2.75e-08 | Spinal meningioma | -0.197 | 0.107 | 0.065 | 0.000 | 30.908 |
| rs72805613 | Type 1 diabetes | 0.098 | 0.018 | 4.04e-08 | Spinal meningioma | -0.048 | 0.079 | 0.547 | 0.000 | 30.096 |
| rs7567242 | Type 1 diabetes | 0.142 | 0.024 | 4.17e-09 | Spinal meningioma | 0.017 | 0.110 | 0.875 | 0.000 | 34.522 |
| rs7903146 | Type 1 diabetes | 0.167 | 0.022 | 1.48e-14 | Spinal meningioma | 0.064 | 0.096 | 0.509 | 0.000 | 59.084 |
| rs9273363 | Type 1 diabetes | 0.730 | 0.020 | 1.00e-200 | Spinal meningioma | -0.170 | 0.086 | 0.049 | 0.003 | 1,402.977 |
| rs10184004 | Type 2 diabetes | -0.003 | 0.000 | 4.40e-15 | Intracranial meningioma | -0.021 | 0.035 | 0.555 | 0.000 | 62.313 |
| rs10228456 | Type 2 diabetes | 0.003 | 0.000 | 1.00e-14 | Intracranial meningioma | -0.002 | 0.034 | 0.959 | 0.000 | 59.345 |
| rs10420309 | Type 2 diabetes | -0.003 | 0.000 | 2.10e-13 | Intracranial meningioma | -0.048 | 0.034 | 0.157 | 0.000 | 54.116 |
| rs10811660 | Type 2 diabetes | -0.006 | 0.001 | 1.20e-31 | Intracranial meningioma | -0.009 | 0.048 | 0.857 | 0.000 | 135.513 |
| rs10830963 | Type 2 diabetes | 0.004 | 0.000 | 1.30e-16 | Intracranial meningioma | 0.006 | 0.035 | 0.861 | 0.000 | 67.388 |
| rs10974438 | Type 2 diabetes | 0.003 | 0.000 | 6.10e-09 | Intracranial meningioma | 0.049 | 0.034 | 0.153 | 0.000 | 34.105 |
| rs11257655 | Type 2 diabetes | 0.004 | 0.001 | 6.60e-14 | Intracranial meningioma | -0.136 | 0.038 | 3.96e-04 | 0.000 | 57.125 |
| rs112674299 | Type 2 diabetes | -0.004 | 0.001 | 3.30e-09 | Intracranial meningioma | 0.009 | 0.041 | 0.826 | 0.000 | 34.355 |
| rs112845979 | Type 2 diabetes | -0.013 | 0.002 | 1.70e-08 | Intracranial meningioma | 0.010 | 0.088 | 0.912 | 0.000 | 31.287 |
| rs115505614 | Type 2 diabetes | 0.008 | 0.001 | 7.30e-16 | Intracranial meningioma | -0.078 | 0.071 | 0.269 | 0.000 | 66.011 |
| rs11602873 | Type 2 diabetes | -0.005 | 0.001 | 5.20e-17 | Intracranial meningioma | 0.006 | 0.039 | 0.885 | 0.000 | 71.537 |
| rs11688682 | Type 2 diabetes | -0.003 | 0.000 | 1.70e-08 | Intracranial meningioma | 0.001 | 0.039 | 0.97 | 0.000 | 31.192 |
| rs1169299 | Type 2 diabetes | 0.002 | 0.000 | 1.90e-08 | Intracranial meningioma | -0.012 | 0.033 | 0.715 | 0.000 | 31.665 |
| rs11720108 | Type 2 diabetes | -0.004 | 0.000 | 1.50e-15 | Intracranial meningioma | 0.020 | 0.044 | 0.655 | 0.000 | 62.729 |
| rs11759026 | Type 2 diabetes | 0.003 | 0.000 | 1.00e-11 | Intracranial meningioma | -0.037 | 0.039 | 0.335 | 0.000 | 47.062 |
| rs1215468 | Type 2 diabetes | -0.004 | 0.000 | 4.10e-19 | Intracranial meningioma | 0.043 | 0.036 | 0.238 | 0.000 | 80.929 |
| rs1260326 | Type 2 diabetes | 0.003 | 0.000 | 3.40e-15 | Intracranial meningioma | 0.002 | 0.035 | 0.95 | 0.000 | 63.823 |
| rs12910361 | Type 2 diabetes | 0.003 | 0.000 | 1.20e-11 | Intracranial meningioma | -0.010 | 0.036 | 0.778 | 0.000 | 45.857 |
| rs12967878 | Type 2 diabetes | 0.003 | 0.000 | 2.70e-11 | Intracranial meningioma | 0.017 | 0.043 | 0.691 | 0.000 | 43.978 |
| rs1320164 | Type 2 diabetes | -0.002 | 0.000 | 2.60e-09 | Intracranial meningioma | 0.001 | 0.033 | 0.981 | 0.000 | 36.484 |
| rs13414381 | Type 2 diabetes | -0.005 | 0.001 | 1.00e-16 | Intracranial meningioma | 0.074 | 0.067 | 0.265 | 0.000 | 69.110 |
| rs1421085 | Type 2 diabetes | 0.005 | 0.000 | 3.10e-29 | Intracranial meningioma | 0.021 | 0.034 | 0.531 | 0.000 | 125.710 |
| rs1496653 | Type 2 diabetes | -0.003 | 0.001 | 1.10e-11 | Intracranial meningioma | 0.009 | 0.036 | 0.8 | 0.000 | 45.313 |
| rs17036160 | Type 2 diabetes | -0.004 | 0.001 | 4.70e-12 | Intracranial meningioma | 0.022 | 0.044 | 0.624 | 0.000 | 46.125 |
| rs17513135 | Type 2 diabetes | 0.003 | 0.000 | 7.30e-11 | Intracranial meningioma | 0.056 | 0.041 | 0.172 | 0.000 | 43.420 |
| rs1800961 | Type 2 diabetes | 0.008 | 0.001 | 1.60e-11 | Intracranial meningioma | -0.102 | 0.080 | 0.202 | 0.000 | 45.466 |
| rs1801212 | Type 2 diabetes | 0.004 | 0.000 | 9.60e-19 | Intracranial meningioma | -0.050 | 0.036 | 0.156 | 0.000 | 75.783 |
| rs2237895 | Type 2 diabetes | 0.004 | 0.000 | 1.80e-23 | Intracranial meningioma | 0.054 | 0.034 | 0.11 | 0.000 | 98.643 |
| rs2258238 | Type 2 diabetes | 0.005 | 0.001 | 1.70e-12 | Intracranial meningioma | -0.102 | 0.063 | 0.106 | 0.000 | 50.826 |
| rs2278524 | Type 2 diabetes | 0.003 | 0.000 | 1.40e-08 | Intracranial meningioma | -0.035 | 0.037 | 0.34 | 0.000 | 31.412 |
| rs2303700 | Type 2 diabetes | -0.003 | 0.000 | 2.40e-09 | Intracranial meningioma | 0.053 | 0.036 | 0.141 | 0.000 | 36.456 |
| rs243018 | Type 2 diabetes | 0.003 | 0.000 | 2.60e-09 | Intracranial meningioma | 0.016 | 0.034 | 0.632 | 0.000 | 35.442 |
| rs2510078 | Type 2 diabetes | 0.003 | 0.000 | 8.60e-09 | Intracranial meningioma | 0.061 | 0.045 | 0.175 | 0.000 | 33.905 |
| rs2793829 | Type 2 diabetes | 0.004 | 0.001 | 2.80e-08 | Intracranial meningioma | -0.035 | 0.048 | 0.465 | 0.000 | 31.323 |
| rs2796441 | Type 2 diabetes | -0.003 | 0.000 | 7.20e-16 | Intracranial meningioma | 0.002 | 0.034 | 0.964 | 0.000 | 64.864 |
| rs28641468 | Type 2 diabetes | 0.003 | 0.000 | 3.60e-08 | Intracranial meningioma | 0.016 | 0.036 | 0.651 | 0.000 | 31.832 |
| rs28746853 | Type 2 diabetes | 0.004 | 0.001 | 7.60e-10 | Intracranial meningioma | -0.064 | 0.038 | 0.089 | 0.000 | 38.156 |
| rs2972143 | Type 2 diabetes | 0.004 | 0.000 | 9.10e-17 | Intracranial meningioma | -0.016 | 0.035 | 0.637 | 0.000 | 67.719 |
| rs3383 | Type 2 diabetes | 0.002 | 0.000 | 1.90e-08 | Intracranial meningioma | -0.009 | 0.034 | 0.798 | 0.000 | 31.095 |
| rs34715063 | Type 2 diabetes | 0.005 | 0.001 | 3.70e-13 | Intracranial meningioma | -0.048 | 0.055 | 0.375 | 0.000 | 53.810 |
| rs34744311 | Type 2 diabetes | -0.005 | 0.000 | 3.30e-27 | Intracranial meningioma | 0.009 | 0.034 | 0.784 | 0.000 | 115.861 |
| rs348330 | Type 2 diabetes | -0.002 | 0.000 | 7.00e-09 | Intracranial meningioma | -0.001 | 0.035 | 0.984 | 0.000 | 33.042 |
| rs3802177 | Type 2 diabetes | -0.005 | 0.000 | 3.40e-24 | Intracranial meningioma | 0.018 | 0.034 | 0.601 | 0.000 | 103.625 |
| rs4287436 | Type 2 diabetes | 0.002 | 0.000 | 5.60e-09 | Intracranial meningioma | -0.068 | 0.034 | 0.048 | 0.000 | 33.140 |
| rs4686471 | Type 2 diabetes | 0.003 | 0.000 | 6.20e-12 | Intracranial meningioma | -0.002 | 0.034 | 0.956 | 0.000 | 45.952 |
| rs4715207 | Type 2 diabetes | 0.004 | 0.001 | 1.80e-11 | Intracranial meningioma | -0.010 | 0.041 | 0.805 | 0.000 | 46.202 |
| rs4729854 | Type 2 diabetes | 0.003 | 0.000 | 3.40e-10 | Intracranial meningioma | -0.006 | 0.034 | 0.856 | 0.000 | 39.689 |
| rs508419 | Type 2 diabetes | 0.004 | 0.000 | 1.70e-15 | Intracranial meningioma | -0.060 | 0.041 | 0.145 | 0.000 | 63.190 |
| rs5213 | Type 2 diabetes | -0.003 | 0.000 | 2.00e-13 | Intracranial meningioma | -0.011 | 0.033 | 0.734 | 0.000 | 53.289 |
| rs6446298 | Type 2 diabetes | 0.003 | 0.000 | 2.40e-09 | Intracranial meningioma | -0.073 | 0.034 | 0.031 | 0.000 | 36.116 |
| rs6885132 | Type 2 diabetes | -0.005 | 0.001 | 2.40e-13 | Intracranial meningioma | -0.056 | 0.052 | 0.286 | 0.000 | 52.985 |
| rs703972 | Type 2 diabetes | -0.003 | 0.000 | 3.20e-15 | Intracranial meningioma | 0.034 | 0.033 | 0.312 | 0.000 | 60.817 |
| rs7274168 | Type 2 diabetes | 0.002 | 0.000 | 2.10e-09 | Intracranial meningioma | 0.106 | 0.035 | 0.002 | 0.000 | 33.868 |
| rs72802365 | Type 2 diabetes | -0.006 | 0.001 | 5.70e-14 | Intracranial meningioma | 0.028 | 0.061 | 0.65 | 0.000 | 57.232 |
| rs7482891 | Type 2 diabetes | -0.003 | 0.000 | 5.90e-11 | Intracranial meningioma | -0.003 | 0.035 | 0.921 | 0.000 | 42.500 |
| rs7501939 | Type 2 diabetes | -0.004 | 0.000 | 1.00e-17 | Intracranial meningioma | -0.047 | 0.036 | 0.199 | 0.000 | 72.470 |
| rs7633675 | Type 2 diabetes | 0.005 | 0.000 | 5.90e-28 | Intracranial meningioma | -0.012 | 0.036 | 0.747 | 0.000 | 117.026 |
| rs76895963 | Type 2 diabetes | -0.019 | 0.002 | 1.90e-32 | Intracranial meningioma | 0.251 | 0.093 | 0.007 | 0.000 | 135.631 |
| rs7756992 | Type 2 diabetes | 0.005 | 0.000 | 1.00e-31 | Intracranial meningioma | -0.029 | 0.035 | 0.418 | 0.000 | 134.309 |
| rs7807512 | Type 2 diabetes | 0.003 | 0.000 | 5.40e-09 | Intracranial meningioma | 0.044 | 0.037 | 0.23 | 0.000 | 34.339 |
| rs7903146 | Type 2 diabetes | 0.014 | 0.000 | 1.00e-200 | Intracranial meningioma | -0.025 | 0.042 | 0.543 | 0.002 | 925.687 |
| rs79687284 | Type 2 diabetes | 0.008 | 0.001 | 1.90e-12 | Intracranial meningioma | -0.079 | 0.128 | 0.539 | 0.000 | 48.420 |
| rs7988007 | Type 2 diabetes | -0.003 | 0.001 | 3.10e-08 | Intracranial meningioma | -0.059 | 0.045 | 0.194 | 0.000 | 29.645 |
| rs8031576 | Type 2 diabetes | -0.003 | 0.000 | 3.30e-09 | Intracranial meningioma | -0.005 | 0.038 | 0.902 | 0.000 | 36.265 |
| rs860262 | Type 2 diabetes | -0.004 | 0.000 | 2.30e-18 | Intracranial meningioma | -0.021 | 0.033 | 0.531 | 0.000 | 76.997 |
| rs9379084 | Type 2 diabetes | -0.005 | 0.001 | 1.20e-15 | Intracranial meningioma | 0.015 | 0.053 | 0.782 | 0.000 | 62.692 |
| rs9686661 | Type 2 diabetes | 0.003 | 0.001 | 1.50e-11 | Intracranial meningioma | 0.074 | 0.049 | 0.131 | 0.000 | 44.354 |
| rs10184004 | Type 2 diabetes | -0.003 | 0.000 | 4.40e-15 | Spinal meningioma | -0.004 | 0.080 | 0.961 | 0.000 | 62.313 |
| rs10228456 | Type 2 diabetes | 0.003 | 0.000 | 1.00e-14 | Spinal meningioma | 0.048 | 0.078 | 0.534 | 0.000 | 59.345 |
| rs10420309 | Type 2 diabetes | -0.003 | 0.000 | 2.10e-13 | Spinal meningioma | -0.038 | 0.078 | 0.625 | 0.000 | 54.116 |
| rs10811660 | Type 2 diabetes | -0.006 | 0.001 | 1.20e-31 | Spinal meningioma | -0.024 | 0.109 | 0.826 | 0.000 | 135.513 |
| rs10830963 | Type 2 diabetes | 0.004 | 0.000 | 1.30e-16 | Spinal meningioma | 0.083 | 0.080 | 0.303 | 0.000 | 67.388 |
| rs10974438 | Type 2 diabetes | 0.003 | 0.000 | 6.10e-09 | Spinal meningioma | 0.013 | 0.080 | 0.875 | 0.000 | 34.105 |
| rs11257655 | Type 2 diabetes | 0.004 | 0.001 | 6.60e-14 | Spinal meningioma | -0.084 | 0.088 | 0.336 | 0.000 | 57.125 |
| rs112674299 | Type 2 diabetes | -0.004 | 0.001 | 3.30e-09 | Spinal meningioma | -0.100 | 0.095 | 0.292 | 0.000 | 34.355 |
| rs112845979 | Type 2 diabetes | -0.013 | 0.002 | 1.70e-08 | Spinal meningioma | -0.242 | 0.202 | 0.23 | 0.000 | 31.287 |
| rs115505614 | Type 2 diabetes | 0.008 | 0.001 | 7.30e-16 | Spinal meningioma | -0.070 | 0.168 | 0.679 | 0.000 | 66.011 |
| rs11602873 | Type 2 diabetes | -0.005 | 0.001 | 5.20e-17 | Spinal meningioma | -0.050 | 0.091 | 0.581 | 0.000 | 71.537 |
| rs11688682 | Type 2 diabetes | -0.003 | 0.000 | 1.70e-08 | Spinal meningioma | 0.013 | 0.090 | 0.888 | 0.000 | 31.192 |
| rs1169299 | Type 2 diabetes | 0.002 | 0.000 | 1.90e-08 | Spinal meningioma | 0.066 | 0.077 | 0.394 | 0.000 | 31.665 |
| rs11720108 | Type 2 diabetes | -0.004 | 0.000 | 1.50e-15 | Spinal meningioma | 0.000 | 0.101 | 0.997 | 0.000 | 62.729 |
| rs11759026 | Type 2 diabetes | 0.003 | 0.000 | 1.00e-11 | Spinal meningioma | -0.138 | 0.090 | 0.124 | 0.000 | 47.062 |
| rs1215468 | Type 2 diabetes | -0.004 | 0.000 | 4.10e-19 | Spinal meningioma | 0.035 | 0.084 | 0.681 | 0.000 | 80.929 |
| rs1260326 | Type 2 diabetes | 0.003 | 0.000 | 3.40e-15 | Spinal meningioma | -0.059 | 0.081 | 0.463 | 0.000 | 63.823 |
| rs12910361 | Type 2 diabetes | 0.003 | 0.000 | 1.20e-11 | Spinal meningioma | 0.079 | 0.083 | 0.342 | 0.000 | 45.857 |
| rs12967878 | Type 2 diabetes | 0.003 | 0.000 | 2.70e-11 | Spinal meningioma | -0.020 | 0.100 | 0.845 | 0.000 | 43.978 |
| rs1320164 | Type 2 diabetes | -0.002 | 0.000 | 2.60e-09 | Spinal meningioma | -0.160 | 0.077 | 0.038 | 0.000 | 36.484 |
| rs13414381 | Type 2 diabetes | -0.005 | 0.001 | 1.00e-16 | Spinal meningioma | -0.064 | 0.154 | 0.679 | 0.000 | 69.110 |
| rs1421085 | Type 2 diabetes | 0.005 | 0.000 | 3.10e-29 | Spinal meningioma | -0.048 | 0.078 | 0.537 | 0.000 | 125.710 |
| rs1496653 | Type 2 diabetes | -0.003 | 0.001 | 1.10e-11 | Spinal meningioma | 0.003 | 0.083 | 0.97 | 0.000 | 45.313 |
| rs17036160 | Type 2 diabetes | -0.004 | 0.001 | 4.70e-12 | Spinal meningioma | 0.129 | 0.102 | 0.207 | 0.000 | 46.125 |
| rs17513135 | Type 2 diabetes | 0.003 | 0.000 | 7.30e-11 | Spinal meningioma | -0.015 | 0.094 | 0.874 | 0.000 | 43.420 |
| rs1800961 | Type 2 diabetes | 0.008 | 0.001 | 1.60e-11 | Spinal meningioma | -0.071 | 0.184 | 0.7 | 0.000 | 45.466 |
| rs1801212 | Type 2 diabetes | 0.004 | 0.000 | 9.60e-19 | Spinal meningioma | 0.009 | 0.082 | 0.91 | 0.000 | 75.783 |
| rs2237895 | Type 2 diabetes | 0.004 | 0.000 | 1.80e-23 | Spinal meningioma | 0.125 | 0.078 | 0.109 | 0.000 | 98.643 |
| rs2258238 | Type 2 diabetes | 0.005 | 0.001 | 1.70e-12 | Spinal meningioma | -0.014 | 0.145 | 0.926 | 0.000 | 50.826 |
| rs2278524 | Type 2 diabetes | 0.003 | 0.000 | 1.40e-08 | Spinal meningioma | 0.095 | 0.085 | 0.263 | 0.000 | 31.412 |
| rs2303700 | Type 2 diabetes | -0.003 | 0.000 | 2.40e-09 | Spinal meningioma | -0.002 | 0.082 | 0.985 | 0.000 | 36.456 |
| rs243018 | Type 2 diabetes | 0.003 | 0.000 | 2.60e-09 | Spinal meningioma | -0.072 | 0.078 | 0.356 | 0.000 | 35.442 |
| rs2510078 | Type 2 diabetes | 0.003 | 0.000 | 8.60e-09 | Spinal meningioma | -0.075 | 0.103 | 0.471 | 0.000 | 33.905 |
| rs2793829 | Type 2 diabetes | 0.004 | 0.001 | 2.80e-08 | Spinal meningioma | -0.065 | 0.112 | 0.561 | 0.000 | 31.323 |
| rs2796441 | Type 2 diabetes | -0.003 | 0.000 | 7.20e-16 | Spinal meningioma | 0.023 | 0.078 | 0.771 | 0.000 | 64.864 |
| rs28641468 | Type 2 diabetes | 0.003 | 0.000 | 3.60e-08 | Spinal meningioma | -0.035 | 0.084 | 0.675 | 0.000 | 31.832 |
| rs28746853 | Type 2 diabetes | 0.004 | 0.001 | 7.60e-10 | Spinal meningioma | -0.163 | 0.088 | 0.063 | 0.000 | 38.156 |
| rs2972143 | Type 2 diabetes | 0.004 | 0.000 | 9.10e-17 | Spinal meningioma | 0.022 | 0.080 | 0.779 | 0.000 | 67.719 |
| rs3383 | Type 2 diabetes | 0.002 | 0.000 | 1.90e-08 | Spinal meningioma | 0.015 | 0.080 | 0.852 | 0.000 | 31.095 |
| rs34715063 | Type 2 diabetes | 0.005 | 0.001 | 3.70e-13 | Spinal meningioma | -0.078 | 0.127 | 0.539 | 0.000 | 53.810 |
| rs34744311 | Type 2 diabetes | -0.005 | 0.000 | 3.30e-27 | Spinal meningioma | 0.089 | 0.079 | 0.257 | 0.000 | 115.861 |
| rs348330 | Type 2 diabetes | -0.002 | 0.000 | 7.00e-09 | Spinal meningioma | -0.052 | 0.081 | 0.522 | 0.000 | 33.042 |
| rs3802177 | Type 2 diabetes | -0.005 | 0.000 | 3.40e-24 | Spinal meningioma | -0.117 | 0.080 | 0.141 | 0.000 | 103.625 |
| rs4287436 | Type 2 diabetes | 0.002 | 0.000 | 5.60e-09 | Spinal meningioma | 0.051 | 0.079 | 0.516 | 0.000 | 33.140 |
| rs4686471 | Type 2 diabetes | 0.003 | 0.000 | 6.20e-12 | Spinal meningioma | -0.096 | 0.079 | 0.227 | 0.000 | 45.952 |
| rs4715207 | Type 2 diabetes | 0.004 | 0.001 | 1.80e-11 | Spinal meningioma | -0.129 | 0.094 | 0.167 | 0.000 | 46.202 |
| rs4729854 | Type 2 diabetes | 0.003 | 0.000 | 3.40e-10 | Spinal meningioma | -0.077 | 0.078 | 0.322 | 0.000 | 39.689 |
| rs508419 | Type 2 diabetes | 0.004 | 0.000 | 1.70e-15 | Spinal meningioma | 0.042 | 0.096 | 0.657 | 0.000 | 63.190 |
| rs5213 | Type 2 diabetes | -0.003 | 0.000 | 2.00e-13 | Spinal meningioma | 0.061 | 0.077 | 0.426 | 0.000 | 53.289 |
| rs6446298 | Type 2 diabetes | 0.003 | 0.000 | 2.40e-09 | Spinal meningioma | -0.058 | 0.078 | 0.458 | 0.000 | 36.116 |
| rs6885132 | Type 2 diabetes | -0.005 | 0.001 | 2.40e-13 | Spinal meningioma | -0.092 | 0.121 | 0.449 | 0.000 | 52.985 |
| rs703972 | Type 2 diabetes | -0.003 | 0.000 | 3.20e-15 | Spinal meningioma | 0.024 | 0.077 | 0.759 | 0.000 | 60.817 |
| rs7274168 | Type 2 diabetes | 0.002 | 0.000 | 2.10e-09 | Spinal meningioma | -0.006 | 0.080 | 0.941 | 0.000 | 33.868 |
| rs72802365 | Type 2 diabetes | -0.006 | 0.001 | 5.70e-14 | Spinal meningioma | 0.085 | 0.143 | 0.552 | 0.000 | 57.232 |
| rs7482891 | Type 2 diabetes | -0.003 | 0.000 | 5.90e-11 | Spinal meningioma | -0.003 | 0.081 | 0.974 | 0.000 | 42.500 |
| rs7501939 | Type 2 diabetes | -0.004 | 0.000 | 1.00e-17 | Spinal meningioma | -0.190 | 0.084 | 0.025 | 0.000 | 72.470 |
| rs7633675 | Type 2 diabetes | 0.005 | 0.000 | 5.90e-28 | Spinal meningioma | 0.056 | 0.083 | 0.504 | 0.000 | 117.026 |
| rs76895963 | Type 2 diabetes | -0.019 | 0.002 | 1.90e-32 | Spinal meningioma | 0.340 | 0.227 | 0.134 | 0.000 | 135.631 |
| rs7756992 | Type 2 diabetes | 0.005 | 0.000 | 1.00e-31 | Spinal meningioma | 0.077 | 0.082 | 0.345 | 0.000 | 134.309 |
| rs7807512 | Type 2 diabetes | 0.003 | 0.000 | 5.40e-09 | Spinal meningioma | -0.045 | 0.085 | 0.6 | 0.000 | 34.339 |
| rs7903146 | Type 2 diabetes | 0.014 | 0.000 | 1.00e-200 | Spinal meningioma | 0.064 | 0.096 | 0.509 | 0.002 | 925.687 |
| rs79687284 | Type 2 diabetes | 0.008 | 0.001 | 1.90e-12 | Spinal meningioma | 0.067 | 0.291 | 0.819 | 0.000 | 48.420 |
| rs7988007 | Type 2 diabetes | -0.003 | 0.001 | 3.10e-08 | Spinal meningioma | 0.032 | 0.104 | 0.758 | 0.000 | 29.645 |
| rs8031576 | Type 2 diabetes | -0.003 | 0.000 | 3.30e-09 | Spinal meningioma | -0.040 | 0.089 | 0.65 | 0.000 | 36.265 |
| rs860262 | Type 2 diabetes | -0.004 | 0.000 | 2.30e-18 | Spinal meningioma | 0.029 | 0.077 | 0.711 | 0.000 | 76.997 |
| rs9379084 | Type 2 diabetes | -0.005 | 0.001 | 1.20e-15 | Spinal meningioma | 0.062 | 0.123 | 0.616 | 0.000 | 62.692 |
| rs9686661 | Type 2 diabetes | 0.003 | 0.001 | 1.50e-11 | Spinal meningioma | 0.082 | 0.112 | 0.466 | 0.000 | 44.354 |
| rs10151436 | Hemoglobin A1c | -0.013 | 0.002 | 3.85e-11 | Intracranial meningioma | 0.019 | 0.055 | 0.734 | 0.000 | 38.321 |
| rs10169706 | Hemoglobin A1c | 0.026 | 0.005 | 1.48e-08 | Intracranial meningioma | 0.097 | 0.064 | 0.129 | 0.000 | 31.947 |
| rs10231021 | Hemoglobin A1c | 0.009 | 0.001 | 8.69e-14 | Intracranial meningioma | 0.003 | 0.034 | 0.927 | 0.000 | 46.869 |
| rs10405535 | Hemoglobin A1c | -0.012 | 0.002 | 6.47e-14 | Intracranial meningioma | -0.029 | 0.039 | 0.465 | 0.000 | 58.140 |
| rs10774624 | Hemoglobin A1c | 0.009 | 0.001 | 4.17e-14 | Intracranial meningioma | -0.023 | 0.034 | 0.501 | 0.000 | 51.177 |
| rs10811661 | Hemoglobin A1c | -0.013 | 0.002 | 1.73e-14 | Intracranial meningioma | -0.009 | 0.048 | 0.856 | 0.000 | 56.691 |
| rs10830963 | Hemoglobin A1c | 0.020 | 0.002 | 1.54e-36 | Intracranial meningioma | 0.006 | 0.035 | 0.861 | 0.001 | 172.482 |
| rs10946402 | Hemoglobin A1c | 0.010 | 0.002 | 1.12e-10 | Intracranial meningioma | -0.073 | 0.045 | 0.105 | 0.000 | 39.847 |
| rs11039154 | Hemoglobin A1c | -0.009 | 0.001 | 3.10e-09 | Intracranial meningioma | 0.088 | 0.043 | 0.039 | 0.000 | 38.617 |
| rs11248914 | Hemoglobin A1c | -0.011 | 0.001 | 1.42e-14 | Intracranial meningioma | 0.001 | 0.038 | 0.983 | 0.000 | 66.305 |
| rs11257655 | Hemoglobin A1c | 0.011 | 0.002 | 1.91e-13 | Intracranial meningioma | -0.136 | 0.038 | 3.96e-04 | 0.000 | 47.265 |
| rs11558471 | Hemoglobin A1c | -0.015 | 0.001 | 3.38e-25 | Intracranial meningioma | 0.021 | 0.034 | 0.55 | 0.001 | 116.330 |
| rs11643024 | Hemoglobin A1c | -0.008 | 0.002 | 7.97e-10 | Intracranial meningioma | -0.051 | 0.038 | 0.171 | 0.000 | 31.360 |
| rs11720108 | Hemoglobin A1c | -0.013 | 0.002 | 2.95e-18 | Intracranial meningioma | 0.020 | 0.044 | 0.655 | 0.001 | 73.959 |
| rs117233107 | Hemoglobin A1c | -0.047 | 0.007 | 8.45e-11 | Intracranial meningioma | 0.323 | 0.142 | 0.023 | 0.000 | 42.611 |
| rs1175549 | Hemoglobin A1c | -0.010 | 0.002 | 7.13e-13 | Intracranial meningioma | 0.018 | 0.037 | 0.637 | 0.000 | 42.684 |
| rs12491937 | Hemoglobin A1c | -0.009 | 0.001 | 1.42e-13 | Intracranial meningioma | -0.013 | 0.034 | 0.708 | 0.000 | 47.928 |
| rs12612492 | Hemoglobin A1c | 0.019 | 0.002 | 1.88e-26 | Intracranial meningioma | -0.074 | 0.057 | 0.195 | 0.001 | 97.904 |
| rs1278769 | Hemoglobin A1c | 0.009 | 0.002 | 5.51e-12 | Intracranial meningioma | -0.014 | 0.039 | 0.713 | 0.000 | 36.804 |
| rs13089972 | Hemoglobin A1c | 0.011 | 0.001 | 1.87e-15 | Intracranial meningioma | 0.062 | 0.034 | 0.068 | 0.000 | 62.861 |
| rs13134327 | Hemoglobin A1c | 0.014 | 0.001 | 2.81e-26 | Intracranial meningioma | 0.005 | 0.035 | 0.89 | 0.001 | 105.794 |
| rs13234131 | Hemoglobin A1c | 0.011 | 0.002 | 2.06e-09 | Intracranial meningioma | -0.074 | 0.050 | 0.14 | 0.000 | 31.922 |
| rs13389076 | Hemoglobin A1c | 0.033 | 0.004 | 3.04e-18 | Intracranial meningioma | 0.045 | 0.080 | 0.572 | 0.001 | 76.331 |
| rs13419763 | Hemoglobin A1c | 0.008 | 0.001 | 5.48e-09 | Intracranial meningioma | -0.030 | 0.035 | 0.394 | 0.000 | 32.653 |
| rs1367173 | Hemoglobin A1c | -0.015 | 0.002 | 1.66e-14 | Intracranial meningioma | 0.057 | 0.064 | 0.377 | 0.000 | 57.759 |
| rs151165 | Hemoglobin A1c | 0.008 | 0.001 | 2.04e-09 | Intracranial meningioma | 0.064 | 0.034 | 0.058 | 0.000 | 31.841 |
| rs1535464 | Hemoglobin A1c | -0.009 | 0.002 | 1.11e-08 | Intracranial meningioma | 0.037 | 0.042 | 0.38 | 0.000 | 25.591 |
| rs174559 | Hemoglobin A1c | -0.011 | 0.001 | 3.31e-13 | Intracranial meningioma | -0.038 | 0.036 | 0.285 | 0.000 | 57.326 |
| rs17476364 | Hemoglobin A1c | -0.086 | 0.002 | 1.00e-200 | Intracranial meningioma | 0.000 | 0.071 | 0.998 | 0.009 | 1,391.595 |
| rs17533945 | Hemoglobin A1c | 0.013 | 0.001 | 1.62e-23 | Intracranial meningioma | -0.034 | 0.033 | 0.306 | 0.001 | 83.591 |
| rs1800562 | Hemoglobin A1c | -0.038 | 0.003 | 2.33e-50 | Intracranial meningioma | -0.062 | 0.089 | 0.482 | 0.001 | 201.217 |
| rs2001945 | Hemoglobin A1c | 0.007 | 0.001 | 6.18e-10 | Intracranial meningioma | 0.022 | 0.033 | 0.517 | 0.000 | 28.171 |
| rs2015803 | Hemoglobin A1c | 0.011 | 0.002 | 2.28e-12 | Intracranial meningioma | -0.039 | 0.037 | 0.294 | 0.000 | 48.999 |
| rs204995 | Hemoglobin A1c | 0.010 | 0.002 | 1.93e-09 | Intracranial meningioma | -0.058 | 0.047 | 0.216 | 0.000 | 29.642 |
| rs2375278 | Hemoglobin A1c | -0.011 | 0.002 | 1.05e-11 | Intracranial meningioma | -0.022 | 0.041 | 0.599 | 0.000 | 43.404 |
| rs2613522 | Hemoglobin A1c | 0.027 | 0.002 | 9.34e-39 | Intracranial meningioma | -0.058 | 0.038 | 0.125 | 0.001 | 165.304 |
| rs267738 | Hemoglobin A1c | -0.011 | 0.002 | 1.14e-11 | Intracranial meningioma | 0.074 | 0.042 | 0.082 | 0.000 | 46.410 |
| rs2908277 | Hemoglobin A1c | 0.017 | 0.002 | 1.29e-18 | Intracranial meningioma | -0.076 | 0.046 | 0.098 | 0.000 | 68.889 |
| rs2971670 | Hemoglobin A1c | 0.032 | 0.002 | 5.10e-88 | Intracranial meningioma | 0.046 | 0.054 | 0.391 | 0.002 | 345.518 |
| rs340882 | Hemoglobin A1c | 0.008 | 0.001 | 1.48e-10 | Intracranial meningioma | -0.012 | 0.034 | 0.722 | 0.000 | 41.751 |
| rs360147 | Hemoglobin A1c | -0.009 | 0.002 | 2.08e-09 | Intracranial meningioma | 0.070 | 0.036 | 0.054 | 0.000 | 32.871 |
| rs3778321 | Hemoglobin A1c | -0.011 | 0.002 | 4.18e-11 | Intracranial meningioma | 0.011 | 0.040 | 0.791 | 0.000 | 43.890 |
| rs3829109 | Hemoglobin A1c | -0.009 | 0.002 | 2.68e-08 | Intracranial meningioma | -0.033 | 0.035 | 0.338 | 0.000 | 32.871 |
| rs452306 | Hemoglobin A1c | -0.010 | 0.001 | 5.51e-13 | Intracranial meningioma | 0.077 | 0.033 | 0.021 | 0.000 | 48.999 |
| rs4727979 | Hemoglobin A1c | -0.012 | 0.002 | 4.60e-08 | Intracranial meningioma | 0.036 | 0.051 | 0.475 | 0.000 | 25.418 |
| rs4737009 | Hemoglobin A1c | 0.023 | 0.002 | 8.29e-56 | Intracranial meningioma | -0.069 | 0.039 | 0.074 | 0.002 | 231.037 |
| rs4760682 | Hemoglobin A1c | 0.016 | 0.002 | 3.20e-20 | Intracranial meningioma | 0.016 | 0.046 | 0.731 | 0.001 | 83.011 |
| rs4980325 | Hemoglobin A1c | 0.011 | 0.001 | 4.70e-14 | Intracranial meningioma | -0.020 | 0.034 | 0.548 | 0.000 | 59.509 |
| rs560887 | Hemoglobin A1c | 0.031 | 0.001 | 5.55e-122 | Intracranial meningioma | -0.035 | 0.036 | 0.328 | 0.003 | 480.856 |
| rs608793 | Hemoglobin A1c | 0.006 | 0.001 | 4.54e-08 | Intracranial meningioma | 0.008 | 0.033 | 0.81 | 0.000 | 25.000 |
| rs61750929 | Hemoglobin A1c | -0.028 | 0.003 | 9.49e-24 | Intracranial meningioma | -0.030 | 0.093 | 0.748 | 0.001 | 95.904 |
| rs6474359 | Hemoglobin A1c | -0.043 | 0.004 | 1.91e-33 | Intracranial meningioma | 0.080 | 0.132 | 0.545 | 0.001 | 126.265 |
| rs651007 | Hemoglobin A1c | 0.011 | 0.002 | 3.28e-15 | Intracranial meningioma | 0.061 | 0.040 | 0.127 | 0.000 | 51.839 |
| rs6798941 | Hemoglobin A1c | 0.009 | 0.002 | 1.49e-08 | Intracranial meningioma | -0.024 | 0.037 | 0.521 | 0.000 | 32.871 |
| rs6804915 | Hemoglobin A1c | -0.011 | 0.001 | 2.76e-16 | Intracranial meningioma | -0.055 | 0.038 | 0.144 | 0.000 | 59.509 |
| rs6877043 | Hemoglobin A1c | -0.009 | 0.001 | 1.99e-10 | Intracranial meningioma | -0.062 | 0.034 | 0.068 | 0.000 | 36.862 |
| rs7042939 | Hemoglobin A1c | -0.010 | 0.001 | 1.50e-15 | Intracranial meningioma | -0.008 | 0.033 | 0.821 | 0.000 | 61.561 |
| rs7127313 | Hemoglobin A1c | 0.007 | 0.001 | 4.85e-08 | Intracranial meningioma | -0.004 | 0.035 | 0.914 | 0.000 | 25.775 |
| rs7190771 | Hemoglobin A1c | 0.009 | 0.001 | 6.02e-11 | Intracranial meningioma | -0.036 | 0.034 | 0.285 | 0.000 | 42.751 |
| rs7198799 | Hemoglobin A1c | 0.008 | 0.001 | 4.76e-09 | Intracranial meningioma | 0.108 | 0.038 | 0.004 | 0.000 | 35.147 |
| rs737092 | Hemoglobin A1c | 0.007 | 0.001 | 7.57e-09 | Intracranial meningioma | -0.006 | 0.033 | 0.862 | 0.000 | 31.532 |
| rs7547793 | Hemoglobin A1c | 0.012 | 0.002 | 6.61e-09 | Intracranial meningioma | 0.013 | 0.045 | 0.774 | 0.000 | 31.573 |
| rs76533333 | Hemoglobin A1c | 0.026 | 0.002 | 2.81e-29 | Intracranial meningioma | 0.014 | 0.080 | 0.858 | 0.001 | 112.358 |
| rs7861647 | Hemoglobin A1c | 0.013 | 0.002 | 4.50e-14 | Intracranial meningioma | 0.018 | 0.043 | 0.667 | 0.000 | 63.999 |
| rs7903146 | Hemoglobin A1c | 0.013 | 0.001 | 1.04e-22 | Intracranial meningioma | -0.025 | 0.042 | 0.543 | 0.001 | 90.249 |
| rs79403657 | Hemoglobin A1c | 0.009 | 0.002 | 2.03e-08 | Intracranial meningioma | -0.042 | 0.042 | 0.312 | 0.000 | 28.027 |
| rs8138197 | Hemoglobin A1c | -0.007 | 0.001 | 3.54e-08 | Intracranial meningioma | 0.004 | 0.033 | 0.909 | 0.000 | 27.188 |
| rs837763 | Hemoglobin A1c | 0.018 | 0.001 | 5.20e-38 | Intracranial meningioma | -0.028 | 0.034 | 0.402 | 0.001 | 183.287 |
| rs855791 | Hemoglobin A1c | -0.019 | 0.001 | 1.34e-56 | Intracranial meningioma | -0.004 | 0.035 | 0.901 | 0.001 | 209.133 |
| rs857725 | Hemoglobin A1c | 0.021 | 0.001 | 5.43e-55 | Intracranial meningioma | -0.028 | 0.035 | 0.425 | 0.002 | 220.732 |
| rs9376090 | Hemoglobin A1c | -0.025 | 0.001 | 1.90e-62 | Intracranial meningioma | 0.073 | 0.035 | 0.038 | 0.002 | 311.266 |
| rs9818758 | Hemoglobin A1c | 0.013 | 0.002 | 1.49e-13 | Intracranial meningioma | 0.030 | 0.041 | 0.468 | 0.000 | 59.380 |
| rs9909940 | Hemoglobin A1c | 0.032 | 0.001 | 1.43e-116 | Intracranial meningioma | 0.028 | 0.038 | 0.462 | 0.004 | 528.993 |
| rs9914988 | Hemoglobin A1c | 0.013 | 0.002 | 4.66e-17 | Intracranial meningioma | 0.052 | 0.043 | 0.221 | 0.000 | 61.034 |
| rs10151436 | Hemoglobin A1c | -0.013 | 0.002 | 3.85e-11 | Spinal meningioma | 0.043 | 0.126 | 0.733 | 0.000 | 38.321 |
| rs10169706 | Hemoglobin A1c | 0.026 | 0.005 | 1.48e-08 | Spinal meningioma | 0.060 | 0.148 | 0.685 | 0.000 | 31.947 |
| rs10231021 | Hemoglobin A1c | 0.009 | 0.001 | 8.69e-14 | Spinal meningioma | 0.031 | 0.078 | 0.686 | 0.000 | 46.869 |
| rs10405535 | Hemoglobin A1c | -0.012 | 0.002 | 6.47e-14 | Spinal meningioma | -0.034 | 0.091 | 0.704 | 0.000 | 58.140 |
| rs10774624 | Hemoglobin A1c | 0.009 | 0.001 | 4.17e-14 | Spinal meningioma | 0.028 | 0.079 | 0.724 | 0.000 | 51.177 |
| rs10811661 | Hemoglobin A1c | -0.013 | 0.002 | 1.73e-14 | Spinal meningioma | -0.024 | 0.109 | 0.826 | 0.000 | 56.691 |
| rs10830963 | Hemoglobin A1c | 0.020 | 0.002 | 1.54e-36 | Spinal meningioma | 0.083 | 0.080 | 0.303 | 0.001 | 172.482 |
| rs10946402 | Hemoglobin A1c | 0.010 | 0.002 | 1.12e-10 | Spinal meningioma | 0.019 | 0.105 | 0.859 | 0.000 | 39.847 |
| rs11039154 | Hemoglobin A1c | -0.009 | 0.001 | 3.10e-09 | Spinal meningioma | -0.020 | 0.098 | 0.838 | 0.000 | 38.617 |
| rs11248914 | Hemoglobin A1c | -0.011 | 0.001 | 1.42e-14 | Spinal meningioma | 0.087 | 0.087 | 0.32 | 0.000 | 66.305 |
| rs11257655 | Hemoglobin A1c | 0.011 | 0.002 | 1.91e-13 | Spinal meningioma | -0.084 | 0.088 | 0.336 | 0.000 | 47.265 |
| rs11558471 | Hemoglobin A1c | -0.015 | 0.001 | 3.38e-25 | Spinal meningioma | -0.109 | 0.079 | 0.169 | 0.001 | 116.330 |
| rs11643024 | Hemoglobin A1c | -0.008 | 0.002 | 7.97e-10 | Spinal meningioma | -0.224 | 0.081 | 0.006 | 0.000 | 31.360 |
| rs11720108 | Hemoglobin A1c | -0.013 | 0.002 | 2.95e-18 | Spinal meningioma | 0.000 | 0.101 | 0.997 | 0.001 | 73.959 |
| rs117233107 | Hemoglobin A1c | -0.047 | 0.007 | 8.45e-11 | Spinal meningioma | 0.016 | 0.321 | 0.959 | 0.000 | 42.611 |
| rs1175549 | Hemoglobin A1c | -0.010 | 0.002 | 7.13e-13 | Spinal meningioma | 0.016 | 0.086 | 0.848 | 0.000 | 42.684 |
| rs12491937 | Hemoglobin A1c | -0.009 | 0.001 | 1.42e-13 | Spinal meningioma | -0.057 | 0.079 | 0.475 | 0.000 | 47.928 |
| rs12612492 | Hemoglobin A1c | 0.019 | 0.002 | 1.88e-26 | Spinal meningioma | -0.229 | 0.132 | 0.082 | 0.001 | 97.904 |
| rs1278769 | Hemoglobin A1c | 0.009 | 0.002 | 5.51e-12 | Spinal meningioma | -0.089 | 0.091 | 0.327 | 0.000 | 36.804 |
| rs13089972 | Hemoglobin A1c | 0.011 | 0.001 | 1.87e-15 | Spinal meningioma | 0.150 | 0.079 | 0.057 | 0.000 | 62.861 |
| rs13134327 | Hemoglobin A1c | 0.014 | 0.001 | 2.81e-26 | Spinal meningioma | -0.064 | 0.082 | 0.433 | 0.001 | 105.794 |
| rs13234131 | Hemoglobin A1c | 0.011 | 0.002 | 2.06e-09 | Spinal meningioma | -0.279 | 0.115 | 0.015 | 0.000 | 31.922 |
| rs13389076 | Hemoglobin A1c | 0.033 | 0.004 | 3.04e-18 | Spinal meningioma | 0.171 | 0.184 | 0.354 | 0.001 | 76.331 |
| rs13419763 | Hemoglobin A1c | 0.008 | 0.001 | 5.48e-09 | Spinal meningioma | -0.024 | 0.082 | 0.77 | 0.000 | 32.653 |
| rs1367173 | Hemoglobin A1c | -0.015 | 0.002 | 1.66e-14 | Spinal meningioma | -0.102 | 0.148 | 0.49 | 0.000 | 57.759 |
| rs151165 | Hemoglobin A1c | 0.008 | 0.001 | 2.04e-09 | Spinal meningioma | 0.060 | 0.079 | 0.447 | 0.000 | 31.841 |
| rs1535464 | Hemoglobin A1c | -0.009 | 0.002 | 1.11e-08 | Spinal meningioma | -0.048 | 0.096 | 0.617 | 0.000 | 25.591 |
| rs174559 | Hemoglobin A1c | -0.011 | 0.001 | 3.31e-13 | Spinal meningioma | 0.030 | 0.083 | 0.721 | 0.000 | 57.326 |
| rs17476364 | Hemoglobin A1c | -0.086 | 0.002 | 1.00e-200 | Spinal meningioma | -0.085 | 0.163 | 0.601 | 0.009 | 1,391.595 |
| rs17533945 | Hemoglobin A1c | 0.013 | 0.001 | 1.62e-23 | Spinal meningioma | -0.018 | 0.077 | 0.812 | 0.001 | 83.591 |
| rs1800562 | Hemoglobin A1c | -0.038 | 0.003 | 2.33e-50 | Spinal meningioma | -0.169 | 0.204 | 0.407 | 0.001 | 201.217 |
| rs2001945 | Hemoglobin A1c | 0.007 | 0.001 | 6.18e-10 | Spinal meningioma | -0.010 | 0.077 | 0.899 | 0.000 | 28.171 |
| rs2015803 | Hemoglobin A1c | 0.011 | 0.002 | 2.28e-12 | Spinal meningioma | -0.116 | 0.086 | 0.177 | 0.000 | 48.999 |
| rs204995 | Hemoglobin A1c | 0.010 | 0.002 | 1.93e-09 | Spinal meningioma | -0.112 | 0.107 | 0.295 | 0.000 | 29.642 |
| rs2375278 | Hemoglobin A1c | -0.011 | 0.002 | 1.05e-11 | Spinal meningioma | -0.053 | 0.095 | 0.577 | 0.000 | 43.404 |
| rs2613522 | Hemoglobin A1c | 0.027 | 0.002 | 9.34e-39 | Spinal meningioma | -0.093 | 0.088 | 0.292 | 0.001 | 165.304 |
| rs267738 | Hemoglobin A1c | -0.011 | 0.002 | 1.14e-11 | Spinal meningioma | 0.160 | 0.098 | 0.101 | 0.000 | 46.410 |
| rs2908277 | Hemoglobin A1c | 0.017 | 0.002 | 1.29e-18 | Spinal meningioma | 0.021 | 0.106 | 0.843 | 0.000 | 68.889 |
| rs2971670 | Hemoglobin A1c | 0.032 | 0.002 | 5.10e-88 | Spinal meningioma | -0.181 | 0.124 | 0.144 | 0.002 | 345.518 |
| rs340882 | Hemoglobin A1c | 0.008 | 0.001 | 1.48e-10 | Spinal meningioma | -0.112 | 0.077 | 0.146 | 0.000 | 41.751 |
| rs360147 | Hemoglobin A1c | -0.009 | 0.002 | 2.08e-09 | Spinal meningioma | 0.134 | 0.085 | 0.113 | 0.000 | 32.871 |
| rs3778321 | Hemoglobin A1c | -0.011 | 0.002 | 4.18e-11 | Spinal meningioma | -0.014 | 0.093 | 0.88 | 0.000 | 43.890 |
| rs3829109 | Hemoglobin A1c | -0.009 | 0.002 | 2.68e-08 | Spinal meningioma | 0.061 | 0.081 | 0.449 | 0.000 | 32.871 |
| rs452306 | Hemoglobin A1c | -0.010 | 0.001 | 5.51e-13 | Spinal meningioma | 0.091 | 0.077 | 0.242 | 0.000 | 48.999 |
| rs4727979 | Hemoglobin A1c | -0.012 | 0.002 | 4.60e-08 | Spinal meningioma | -0.085 | 0.118 | 0.47 | 0.000 | 25.418 |
| rs4737009 | Hemoglobin A1c | 0.023 | 0.002 | 8.29e-56 | Spinal meningioma | -0.022 | 0.089 | 0.802 | 0.002 | 231.037 |
| rs4760682 | Hemoglobin A1c | 0.016 | 0.002 | 3.20e-20 | Spinal meningioma | -0.083 | 0.105 | 0.432 | 0.001 | 83.011 |
| rs4980325 | Hemoglobin A1c | 0.011 | 0.001 | 4.70e-14 | Spinal meningioma | -0.036 | 0.078 | 0.647 | 0.000 | 59.509 |
| rs560887 | Hemoglobin A1c | 0.031 | 0.001 | 5.55e-122 | Spinal meningioma | -0.052 | 0.084 | 0.537 | 0.003 | 480.856 |
| rs608793 | Hemoglobin A1c | 0.006 | 0.001 | 4.54e-08 | Spinal meningioma | 0.063 | 0.077 | 0.414 | 0.000 | 25.000 |
| rs61750929 | Hemoglobin A1c | -0.028 | 0.003 | 9.49e-24 | Spinal meningioma | 0.318 | 0.218 | 0.145 | 0.001 | 95.904 |
| rs6474359 | Hemoglobin A1c | -0.043 | 0.004 | 1.91e-33 | Spinal meningioma | -0.050 | 0.302 | 0.87 | 0.001 | 126.265 |
| rs651007 | Hemoglobin A1c | 0.011 | 0.002 | 3.28e-15 | Spinal meningioma | 0.203 | 0.092 | 0.027 | 0.000 | 51.839 |
| rs6798941 | Hemoglobin A1c | 0.009 | 0.002 | 1.49e-08 | Spinal meningioma | -0.105 | 0.087 | 0.223 | 0.000 | 32.871 |
| rs6804915 | Hemoglobin A1c | -0.011 | 0.001 | 2.76e-16 | Spinal meningioma | -0.064 | 0.087 | 0.461 | 0.000 | 59.509 |
| rs6877043 | Hemoglobin A1c | -0.009 | 0.001 | 1.99e-10 | Spinal meningioma | -0.100 | 0.079 | 0.207 | 0.000 | 36.862 |
| rs7042939 | Hemoglobin A1c | -0.010 | 0.001 | 1.50e-15 | Spinal meningioma | -0.088 | 0.077 | 0.254 | 0.000 | 61.561 |
| rs7127313 | Hemoglobin A1c | 0.007 | 0.001 | 4.85e-08 | Spinal meningioma | -0.140 | 0.081 | 0.085 | 0.000 | 25.775 |
| rs7190771 | Hemoglobin A1c | 0.009 | 0.001 | 6.02e-11 | Spinal meningioma | 0.034 | 0.077 | 0.662 | 0.000 | 42.751 |
| rs7198799 | Hemoglobin A1c | 0.008 | 0.001 | 4.76e-09 | Spinal meningioma | 0.060 | 0.090 | 0.501 | 0.000 | 35.147 |
| rs737092 | Hemoglobin A1c | 0.007 | 0.001 | 7.57e-09 | Spinal meningioma | -0.220 | 0.075 | 0.003 | 0.000 | 31.532 |
| rs7547793 | Hemoglobin A1c | 0.012 | 0.002 | 6.61e-09 | Spinal meningioma | 0.170 | 0.103 | 0.097 | 0.000 | 31.573 |
| rs76533333 | Hemoglobin A1c | 0.026 | 0.002 | 2.81e-29 | Spinal meningioma | 0.229 | 0.184 | 0.214 | 0.001 | 112.358 |
| rs7861647 | Hemoglobin A1c | 0.013 | 0.002 | 4.50e-14 | Spinal meningioma | -0.009 | 0.100 | 0.924 | 0.000 | 63.999 |
| rs7903146 | Hemoglobin A1c | 0.013 | 0.001 | 1.04e-22 | Spinal meningioma | 0.064 | 0.096 | 0.509 | 0.001 | 90.249 |
| rs79403657 | Hemoglobin A1c | 0.009 | 0.002 | 2.03e-08 | Spinal meningioma | 0.037 | 0.096 | 0.697 | 0.000 | 28.027 |
| rs8138197 | Hemoglobin A1c | -0.007 | 0.001 | 3.54e-08 | Spinal meningioma | -0.030 | 0.077 | 0.702 | 0.000 | 27.188 |
| rs837763 | Hemoglobin A1c | 0.018 | 0.001 | 5.20e-38 | Spinal meningioma | -0.093 | 0.077 | 0.23 | 0.001 | 183.287 |
| rs855791 | Hemoglobin A1c | -0.019 | 0.001 | 1.34e-56 | Spinal meningioma | 0.058 | 0.081 | 0.475 | 0.001 | 209.133 |
| rs857725 | Hemoglobin A1c | 0.021 | 0.001 | 5.43e-55 | Spinal meningioma | 0.090 | 0.081 | 0.269 | 0.002 | 220.732 |
| rs9376090 | Hemoglobin A1c | -0.025 | 0.001 | 1.90e-62 | Spinal meningioma | 0.141 | 0.082 | 0.085 | 0.002 | 311.266 |
| rs9818758 | Hemoglobin A1c | 0.013 | 0.002 | 1.49e-13 | Spinal meningioma | 0.059 | 0.095 | 0.532 | 0.000 | 59.380 |
| rs9909940 | Hemoglobin A1c | 0.032 | 0.001 | 1.43e-116 | Spinal meningioma | -0.100 | 0.087 | 0.251 | 0.004 | 528.993 |
| rs9914988 | Hemoglobin A1c | 0.013 | 0.002 | 4.66e-17 | Spinal meningioma | -0.060 | 0.099 | 0.545 | 0.000 | 61.034 |
| rs10248619 | FG | -0.019 | 0.002 | 5.19e-16 | Intracranial meningioma | -0.041 | 0.035 | 0.242 | 0.001 | 63.601 |
| rs10758593 | FG | 0.015 | 0.002 | 1.09e-13 | Intracranial meningioma | 0.044 | 0.034 | 0.188 | 0.000 | 54.620 |
| rs10830963 | FG | 0.077 | 0.002 | 1.00e-200 | Intracranial meningioma | 0.006 | 0.035 | 0.861 | 0.011 | 1,121.767 |
| rs10885117 | FG | 0.028 | 0.004 | 3.81e-15 | Intracranial meningioma | 0.038 | 0.057 | 0.505 | 0.001 | 58.979 |
| rs10965250 | FG | -0.026 | 0.003 | 1.21e-20 | Intracranial meningioma | -0.001 | 0.048 | 0.991 | 0.001 | 84.796 |
| rs11558471 | FG | -0.029 | 0.002 | 7.60e-44 | Intracranial meningioma | 0.021 | 0.034 | 0.55 | 0.002 | 185.157 |
| rs11603334 | FG | -0.026 | 0.003 | 2.49e-23 | Intracranial meningioma | 0.000 | 0.039 | 0.998 | 0.001 | 96.319 |
| rs11619319 | FG | 0.020 | 0.002 | 1.69e-17 | Intracranial meningioma | 0.035 | 0.038 | 0.36 | 0.001 | 69.567 |
| rs11708067 | FG | -0.024 | 0.002 | 4.21e-23 | Intracranial meningioma | 0.035 | 0.044 | 0.435 | 0.001 | 101.458 |
| rs12055786 | FG | 0.014 | 0.003 | 2.71e-08 | Intracranial meningioma | 0.005 | 0.034 | 0.886 | 0.000 | 28.461 |
| rs1260326 | FG | 0.029 | 0.002 | 5.86e-45 | Intracranial meningioma | 0.002 | 0.035 | 0.95 | 0.002 | 194.383 |
| rs16851397 | FG | -0.030 | 0.005 | 1.51e-09 | Intracranial meningioma | 0.084 | 0.091 | 0.357 | 0.000 | 35.256 |
| rs17265513 | FG | 0.017 | 0.002 | 1.57e-11 | Intracranial meningioma | 0.020 | 0.037 | 0.589 | 0.000 | 49.748 |
| rs174550 | FG | -0.021 | 0.002 | 7.30e-23 | Intracranial meningioma | -0.048 | 0.034 | 0.154 | 0.001 | 92.585 |
| rs17776811 | FG | -0.012 | 0.002 | 5.41e-10 | Intracranial meningioma | 0.003 | 0.034 | 0.923 | 0.000 | 34.844 |
| rs1886686 | FG | 0.014 | 0.002 | 1.80e-11 | Intracranial meningioma | -0.006 | 0.035 | 0.865 | 0.000 | 40.396 |
| rs2191349 | FG | 0.026 | 0.002 | 2.31e-38 | Intracranial meningioma | -0.001 | 0.034 | 0.979 | 0.002 | 165.734 |
| rs2206734 | FG | 0.019 | 0.003 | 1.22e-12 | Intracranial meningioma | -0.077 | 0.044 | 0.08 | 0.000 | 50.113 |
| rs2236418 | FG | -0.018 | 0.003 | 1.97e-11 | Intracranial meningioma | 0.040 | 0.048 | 0.41 | 0.000 | 44.991 |
| rs326214 | FG | -0.017 | 0.002 | 7.01e-15 | Intracranial meningioma | 0.011 | 0.034 | 0.741 | 0.001 | 59.037 |
| rs340874 | FG | 0.015 | 0.002 | 3.71e-12 | Intracranial meningioma | 0.012 | 0.034 | 0.726 | 0.000 | 53.749 |
| rs35742417 | FG | -0.021 | 0.003 | 6.23e-18 | Intracranial meningioma | 0.008 | 0.040 | 0.84 | 0.001 | 67.843 |
| rs4607517 | FG | 0.062 | 0.003 | 9.32e-114 | Intracranial meningioma | 0.046 | 0.054 | 0.396 | 0.004 | 500.907 |
| rs479661 | FG | -0.019 | 0.003 | 8.29e-13 | Intracranial meningioma | 0.031 | 0.050 | 0.543 | 0.000 | 47.706 |
| rs5400 | FG | -0.023 | 0.003 | 1.80e-15 | Intracranial meningioma | 0.020 | 0.049 | 0.684 | 0.001 | 60.042 |
| rs560887 | FG | 0.079 | 0.002 | 1.00e-200 | Intracranial meningioma | -0.035 | 0.036 | 0.328 | 0.012 | 1,210.089 |
| rs60980157 | FG | -0.016 | 0.002 | 3.24e-11 | Intracranial meningioma | -0.017 | 0.037 | 0.635 | 0.000 | 40.753 |
| rs6113722 | FG | -0.043 | 0.005 | 3.42e-20 | Intracranial meningioma | -0.060 | 0.087 | 0.492 | 0.001 | 76.625 |
| rs6235 | FG | -0.022 | 0.002 | 1.27e-22 | Intracranial meningioma | -0.006 | 0.037 | 0.873 | 0.001 | 91.697 |
| rs635634 | FG | 0.018 | 0.003 | 5.80e-11 | Intracranial meningioma | 0.073 | 0.042 | 0.081 | 0.000 | 42.370 |
| rs701865 | FG | -0.012 | 0.002 | 2.19e-08 | Intracranial meningioma | -0.056 | 0.036 | 0.116 | 0.000 | 29.099 |
| rs7172432 | FG | -0.022 | 0.002 | 1.92e-27 | Intracranial meningioma | -0.005 | 0.034 | 0.872 | 0.001 | 118.654 |
| rs7578597 | FG | -0.023 | 0.004 | 2.07e-10 | Intracranial meningioma | 0.054 | 0.074 | 0.462 | 0.000 | 38.649 |
| rs76817627 | FG | -0.037 | 0.006 | 4.52e-11 | Intracranial meningioma | -0.050 | 0.110 | 0.648 | 0.000 | 40.270 |
| rs7903146 | FG | 0.022 | 0.002 | 7.34e-22 | Intracranial meningioma | -0.025 | 0.042 | 0.543 | 0.001 | 96.581 |
| rs7945565 | FG | -0.021 | 0.002 | 2.60e-23 | Intracranial meningioma | 0.031 | 0.033 | 0.347 | 0.001 | 102.871 |
| rs881858 | FG | -0.013 | 0.002 | 6.54e-10 | Intracranial meningioma | -0.070 | 0.036 | 0.051 | 0.000 | 37.086 |
| rs9987289 | FG | -0.028 | 0.004 | 6.08e-15 | Intracranial meningioma | 0.069 | 0.047 | 0.146 | 0.001 | 60.134 |
| rs10248619 | FG | -0.019 | 0.002 | 5.19e-16 | Spinal meningioma | -0.038 | 0.081 | 0.636 | 0.001 | 63.601 |
| rs10758593 | FG | 0.015 | 0.002 | 1.09e-13 | Spinal meningioma | -0.006 | 0.078 | 0.934 | 0.000 | 54.620 |
| rs10830963 | FG | 0.077 | 0.002 | 1.00e-200 | Spinal meningioma | 0.083 | 0.080 | 0.303 | 0.011 | 1,121.767 |
| rs10885117 | FG | 0.028 | 0.004 | 3.81e-15 | Spinal meningioma | -0.020 | 0.133 | 0.881 | 0.001 | 58.979 |
| rs10965250 | FG | -0.026 | 0.003 | 1.21e-20 | Spinal meningioma | -0.017 | 0.109 | 0.877 | 0.001 | 84.796 |
| rs11558471 | FG | -0.029 | 0.002 | 7.60e-44 | Spinal meningioma | -0.109 | 0.079 | 0.169 | 0.002 | 185.157 |
| rs11603334 | FG | -0.026 | 0.003 | 2.49e-23 | Spinal meningioma | -0.070 | 0.091 | 0.443 | 0.001 | 96.319 |
| rs11619319 | FG | 0.020 | 0.002 | 1.69e-17 | Spinal meningioma | -0.018 | 0.089 | 0.837 | 0.001 | 69.567 |
| rs11708067 | FG | -0.024 | 0.002 | 4.21e-23 | Spinal meningioma | 0.027 | 0.103 | 0.796 | 0.001 | 101.458 |
| rs12055786 | FG | 0.014 | 0.003 | 2.71e-08 | Spinal meningioma | 0.112 | 0.077 | 0.15 | 0.000 | 28.461 |
| rs1260326 | FG | 0.029 | 0.002 | 5.86e-45 | Spinal meningioma | -0.059 | 0.081 | 0.463 | 0.002 | 194.383 |
| rs16851397 | FG | -0.030 | 0.005 | 1.51e-09 | Spinal meningioma | -0.310 | 0.212 | 0.144 | 0.000 | 35.256 |
| rs17265513 | FG | 0.017 | 0.002 | 1.57e-11 | Spinal meningioma | -0.040 | 0.086 | 0.642 | 0.000 | 49.748 |
| rs174550 | FG | -0.021 | 0.002 | 7.30e-23 | Spinal meningioma | 0.024 | 0.078 | 0.756 | 0.001 | 92.585 |
| rs17776811 | FG | -0.012 | 0.002 | 5.41e-10 | Spinal meningioma | -0.052 | 0.079 | 0.506 | 0.000 | 34.844 |
| rs1886686 | FG | 0.014 | 0.002 | 1.80e-11 | Spinal meningioma | 0.002 | 0.081 | 0.98 | 0.000 | 40.396 |
| rs2191349 | FG | 0.026 | 0.002 | 2.31e-38 | Spinal meningioma | 0.050 | 0.078 | 0.521 | 0.002 | 165.734 |
| rs2206734 | FG | 0.019 | 0.003 | 1.22e-12 | Spinal meningioma | 0.005 | 0.102 | 0.959 | 0.000 | 50.113 |
| rs2236418 | FG | -0.018 | 0.003 | 1.97e-11 | Spinal meningioma | 0.049 | 0.112 | 0.661 | 0.000 | 44.991 |
| rs326214 | FG | -0.017 | 0.002 | 7.01e-15 | Spinal meningioma | -0.020 | 0.079 | 0.8 | 0.001 | 59.037 |
| rs340874 | FG | 0.015 | 0.002 | 3.71e-12 | Spinal meningioma | -0.057 | 0.078 | 0.464 | 0.000 | 53.749 |
| rs35742417 | FG | -0.021 | 0.003 | 6.23e-18 | Spinal meningioma | -0.013 | 0.093 | 0.891 | 0.001 | 67.843 |
| rs4607517 | FG | 0.062 | 0.003 | 9.32e-114 | Spinal meningioma | -0.182 | 0.124 | 0.144 | 0.004 | 500.907 |
| rs479661 | FG | -0.019 | 0.003 | 8.29e-13 | Spinal meningioma | -0.037 | 0.116 | 0.75 | 0.000 | 47.706 |
| rs5400 | FG | -0.023 | 0.003 | 1.80e-15 | Spinal meningioma | 0.068 | 0.114 | 0.548 | 0.001 | 60.042 |
| rs560887 | FG | 0.079 | 0.002 | 1.00e-200 | Spinal meningioma | -0.052 | 0.084 | 0.537 | 0.012 | 1,210.089 |
| rs60980157 | FG | -0.016 | 0.002 | 3.24e-11 | Spinal meningioma | 0.036 | 0.084 | 0.669 | 0.000 | 40.753 |
| rs6113722 | FG | -0.043 | 0.005 | 3.42e-20 | Spinal meningioma | 0.114 | 0.204 | 0.575 | 0.001 | 76.625 |
| rs6235 | FG | -0.022 | 0.002 | 1.27e-22 | Spinal meningioma | 0.147 | 0.085 | 0.085 | 0.001 | 91.697 |
| rs635634 | FG | 0.018 | 0.003 | 5.80e-11 | Spinal meningioma | 0.226 | 0.095 | 0.018 | 0.000 | 42.370 |
| rs701865 | FG | -0.012 | 0.002 | 2.19e-08 | Spinal meningioma | 0.035 | 0.082 | 0.673 | 0.000 | 29.099 |
| rs7172432 | FG | -0.022 | 0.002 | 1.92e-27 | Spinal meningioma | -0.023 | 0.078 | 0.766 | 0.001 | 118.654 |
| rs7578597 | FG | -0.023 | 0.004 | 2.07e-10 | Spinal meningioma | 0.016 | 0.169 | 0.924 | 0.000 | 38.649 |
| rs76817627 | FG | -0.037 | 0.006 | 4.52e-11 | Spinal meningioma | -0.095 | 0.249 | 0.702 | 0.000 | 40.270 |
| rs7903146 | FG | 0.022 | 0.002 | 7.34e-22 | Spinal meningioma | 0.064 | 0.096 | 0.509 | 0.001 | 96.581 |
| rs7945565 | FG | -0.021 | 0.002 | 2.60e-23 | Spinal meningioma | 0.030 | 0.077 | 0.694 | 0.001 | 102.871 |
| rs881858 | FG | -0.013 | 0.002 | 6.54e-10 | Spinal meningioma | -0.022 | 0.083 | 0.789 | 0.000 | 37.086 |
| rs9987289 | FG | -0.028 | 0.004 | 6.08e-15 | Spinal meningioma | 0.148 | 0.109 | 0.176 | 0.001 | 60.134 |
| rs11708067 | 2hGlu | -0.088 | 0.011 | 2.45e-16 | Intracranial meningioma | 0.035 | 0.044 | 0.435 | 0.001 | 66.264 |
| rs1260326 | 2hGlu | -0.068 | 0.009 | 1.65e-14 | Intracranial meningioma | 0.002 | 0.035 | 0.95 | 0.001 | 54.308 |
| rs1800437 | 2hGlu | 0.100 | 0.011 | 1.74e-21 | Intracranial meningioma | 0.006 | 0.038 | 0.868 | 0.002 | 84.696 |
| rs2126259 | 2hGlu | 0.079 | 0.014 | 3.29e-09 | Intracranial meningioma | 0.079 | 0.046 | 0.085 | 0.001 | 32.704 |
| rs2549782 | 2hGlu | -0.056 | 0.009 | 3.31e-10 | Intracranial meningioma | -0.069 | 0.033 | 0.038 | 0.001 | 39.888 |
| rs2650000 | 2hGlu | -0.052 | 0.009 | 5.01e-09 | Intracranial meningioma | 0.002 | 0.034 | 0.948 | 0.001 | 31.768 |
| rs2943641 | 2hGlu | 0.050 | 0.009 | 3.08e-08 | Intracranial meningioma | -0.016 | 0.035 | 0.641 | 0.001 | 30.098 |
| rs4402960 | 2hGlu | 0.052 | 0.010 | 2.51e-08 | Intracranial meningioma | -0.012 | 0.036 | 0.744 | 0.001 | 29.635 |
| rs507666 | 2hGlu | 0.061 | 0.011 | 2.45e-08 | Intracranial meningioma | -0.073 | 0.042 | 0.081 | 0.001 | 29.201 |
| rs6975024 | 2hGlu | 0.103 | 0.013 | 4.58e-17 | Intracranial meningioma | 0.040 | 0.054 | 0.461 | 0.001 | 67.506 |
| rs7178424 | 2hGlu | 0.058 | 0.009 | 4.71e-11 | Intracranial meningioma | -0.024 | 0.035 | 0.497 | 0.001 | 40.596 |
| rs72839768 | 2hGlu | 0.201 | 0.030 | 1.63e-11 | Intracranial meningioma | -0.007 | 0.118 | 0.955 | 0.001 | 43.659 |
| rs7903146 | 2hGlu | 0.087 | 0.010 | 5.84e-19 | Intracranial meningioma | -0.025 | 0.042 | 0.543 | 0.001 | 70.321 |
| rs11708067 | 2hGlu | -0.088 | 0.011 | 2.45e-16 | Spinal meningioma | 0.027 | 0.103 | 0.796 | 0.001 | 66.264 |
| rs1260326 | 2hGlu | -0.068 | 0.009 | 1.65e-14 | Spinal meningioma | -0.059 | 0.081 | 0.463 | 0.001 | 54.308 |
| rs1800437 | 2hGlu | 0.100 | 0.011 | 1.74e-21 | Spinal meningioma | 0.028 | 0.088 | 0.752 | 0.002 | 84.696 |
| rs2126259 | 2hGlu | 0.079 | 0.014 | 3.29e-09 | Spinal meningioma | 0.154 | 0.105 | 0.144 | 0.001 | 32.704 |
| rs2549782 | 2hGlu | -0.056 | 0.009 | 3.31e-10 | Spinal meningioma | 0.145 | 0.077 | 0.06 | 0.001 | 39.888 |
| rs2650000 | 2hGlu | -0.052 | 0.009 | 5.01e-09 | Spinal meningioma | -0.101 | 0.079 | 0.2 | 0.001 | 31.768 |
| rs2943641 | 2hGlu | 0.050 | 0.009 | 3.08e-08 | Spinal meningioma | 0.025 | 0.080 | 0.752 | 0.001 | 30.098 |
| rs4402960 | 2hGlu | 0.052 | 0.010 | 2.51e-08 | Spinal meningioma | 0.056 | 0.083 | 0.504 | 0.001 | 29.635 |
| rs507666 | 2hGlu | 0.061 | 0.011 | 2.45e-08 | Spinal meningioma | -0.226 | 0.095 | 0.018 | 0.001 | 29.201 |
| rs6975024 | 2hGlu | 0.103 | 0.013 | 4.58e-17 | Spinal meningioma | -0.167 | 0.124 | 0.18 | 0.001 | 67.506 |
| rs7178424 | 2hGlu | 0.058 | 0.009 | 4.71e-11 | Spinal meningioma | -0.019 | 0.082 | 0.812 | 0.001 | 40.596 |
| rs72839768 | 2hGlu | 0.201 | 0.030 | 1.63e-11 | Spinal meningioma | -0.511 | 0.271 | 0.059 | 0.001 | 43.659 |
| rs7903146 | 2hGlu | 0.087 | 0.010 | 5.84e-19 | Spinal meningioma | 0.064 | 0.096 | 0.509 | 0.001 | 70.321 |
| rs12704053 | Intracranial meningioma | 0.234 | 0.036 | 6.59e-11 | Type 1 diabetes | 0.004 | 0.018 | 0.812 | 0.000 | 42.637 |
| rs58742727 | Intracranial meningioma | -0.425 | 0.065 | 6.76e-11 | Type 1 diabetes | -0.013 | 0.037 | 0.735 | 0.000 | 42.587 |
| rs72878024 | Intracranial meningioma | -0.480 | 0.070 | 7.61e-12 | Type 1 diabetes | -0.016 | 0.035 | 0.645 | 0.000 | 46.864 |
| rs12704053 | Intracranial meningioma | 0.234 | 0.036 | 6.59e-11 | Type 2 diabetes | 0.000 | 0.000 | 0.38 | 0.000 | 42.637 |
| rs58742727 | Intracranial meningioma | -0.425 | 0.065 | 6.76e-11 | Type 2 diabetes | -0.001 | 0.002 | 0.48 | 0.000 | 42.587 |
| rs72878024 | Intracranial meningioma | -0.480 | 0.070 | 7.61e-12 | Type 2 diabetes | 0.000 | 0.001 | 0.67 | 0.000 | 46.864 |
| rs12704053 | Intracranial meningioma | 0.234 | 0.036 | 6.59e-11 | Hemoglobin A1c | 0.002 | 0.002 | 0.236 | 0.000 | 42.637 |
| rs58742727 | Intracranial meningioma | -0.425 | 0.065 | 6.76e-11 | Hemoglobin A1c | 0.004 | 0.005 | 0.465 | 0.000 | 42.587 |
| rs72878024 | Intracranial meningioma | -0.480 | 0.070 | 7.61e-12 | Hemoglobin A1c | 0.012 | 0.003 | 1.69e-04 | 0.000 | 46.864 |
| rs72878024 | Intracranial meningioma | -0.480 | 0.070 | 7.61e-12 | FG | 0.002 | 0.004 | 0.475 | 0.000 | 46.864 |
| rs72878024 | Intracranial meningioma | -0.480 | 0.070 | 7.61e-12 | 2hGlu | 0.027 | 0.020 | 0.143 | 0.000 | 46.864 |

**Supplementary Table S3.** MR estimates for the causal effect of diabetes and meningioma.

| exposure | outcome | method | Beta | SE | P-value | egger_intercept | se_pleio | pval_pleio | Q_MR_Egger | Q_df_MR_Egger | Q_pval_MR_Egger | Q_ivw | Q_df_ivw | Q_pval_ivw |
| --- | --- | --- | --- | --- | --- | --- | --- | --- | --- | --- | --- | --- | --- | --- |
| Type 1 diabetes | Intracranial meningioma | Inverse variance weighted | 0.033 | 0.034 | 0.334 | 0.042 | 0.016 | 0.020 | 13.281 | 16 | 0.652 | 19.907 | 17 | 0.279 |
| Type 1 diabetes | Intracranial meningioma | MR-Egger | -0.072 | 0.051 | 0.182 | 0.042 | 0.016 | 0.020 | 13.281 | 16 | 0.652 | 19.907 | 17 | 0.279 |
| Type 1 diabetes | Intracranial meningioma | Maximum likelihood | 0.033 | 0.031 | 0.293 | 0.042 | 0.016 | 0.020 | 13.281 | 16 | 0.652 | 19.907 | 17 | 0.279 |
| Type 1 diabetes | Intracranial meningioma | Weighted median | 0.000 | 0.048 | 0.999 | 0.042 | 0.016 | 0.020 | 13.281 | 16 | 0.652 | 19.907 | 17 | 0.279 |
| Type 1 diabetes | Spinal meningioma | Inverse variance weighted | -0.033 | 0.072 | 0.648 | 0.071 | 0.038 | 0.077 | 9.885 | 16 | 0.873 | 13.452 | 17 | 0.705 |
| Type 1 diabetes | Spinal meningioma | MR-Egger | -0.210 | 0.118 | 0.095 | 0.071 | 0.038 | 0.077 | 9.885 | 16 | 0.873 | 13.452 | 17 | 0.705 |
| Type 1 diabetes | Spinal meningioma | Maximum likelihood | -0.033 | 0.073 | 0.649 | 0.071 | 0.038 | 0.077 | 9.885 | 16 | 0.873 | 13.452 | 17 | 0.705 |
| Type 1 diabetes | Spinal meningioma | Weighted median | -0.027 | 0.103 | 0.794 | 0.071 | 0.038 | 0.077 | 9.885 | 16 | 0.873 | 13.452 | 17 | 0.705 |
| Type 2 diabetes | Intracranial meningioma | Inverse variance weighted | -2.594 | 1.306 | 0.047 | 0.021 | 0.012 | 0.090 | 73.459 | 62 | 0.151 | 76.975 | 63 | 0.111 |
| Type 2 diabetes | Intracranial meningioma | MR-Egger | -7.182 | 2.958 | 0.018 | 0.021 | 0.012 | 0.090 | 73.459 | 62 | 0.151 | 76.975 | 63 | 0.111 |
| Type 2 diabetes | Intracranial meningioma | Maximum likelihood | -2.638 | 1.192 | 0.027 | 0.021 | 0.012 | 0.090 | 73.459 | 62 | 0.151 | 76.975 | 63 | 0.111 |
| Type 2 diabetes | Intracranial meningioma | Weighted median | -1.946 | 1.995 | 0.329 | 0.021 | 0.012 | 0.090 | 73.459 | 62 | 0.151 | 76.975 | 63 | 0.111 |
| Type 2 diabetes | Spinal meningioma | Inverse variance weighted | 1.067 | 2.737 | 0.697 | 0.010 | 0.026 | 0.704 | 46.298 | 62 | 0.932 | 46.443 | 63 | 0.942 |
| Type 2 diabetes | Spinal meningioma | MR Egger | -1.110 | 6.329 | 0.861 | 0.010 | 0.026 | 0.704 | 46.298 | 62 | 0.932 | 46.443 | 63 | 0.942 |
| Type 2 diabetes | Spinal meningioma | Maximum likelihood | 1.083 | 2.754 | 0.694 | 0.010 | 0.026 | 0.704 | 46.298 | 62 | 0.932 | 46.443 | 63 | 0.942 |
| Type 2 diabetes | Spinal meningioma | Weighted median | 3.914 | 4.499 | 0.384 | 0.010 | 0.026 | 0.704 | 46.298 | 62 | 0.932 | 46.443 | 63 | 0.942 |
| Hemoglobin A1c | Intracranial meningioma | Inverse variance weighted | -0.625 | 0.359 | 0.082 | -0.002 | 0.011 | 0.840 | 97.789 | 69 | 0.013 | 97.847 | 70 | 0.016 |
| Hemoglobin A1c | Intracranial meningioma | MR Egger | -0.504 | 0.699 | 0.473 | -0.002 | 0.011 | 0.840 | 97.789 | 69 | 0.013 | 97.847 | 70 | 0.016 |
| Hemoglobin A1c | Intracranial meningioma | Maximum likelihood | -0.628 | 0.306 | 0.040 | -0.002 | 0.011 | 0.840 | 97.789 | 69 | 0.013 | 97.847 | 70 | 0.016 |
| Hemoglobin A1c | Intracranial meningioma | Weighted median | -0.050 | 0.499 | 0.921 | -0.002 | 0.011 | 0.840 | 97.789 | 69 | 0.013 | 97.847 | 70 | 0.016 |
| Hemoglobin A1c | Spinal meningioma | Inverse variance weighted | -0.632 | 0.768 | 0.410 | 0.002 | 0.023 | 0.917 | 84.029 | 69 | 0.105 | 84.042 | 70 | 0.121 |
| Hemoglobin A1c | Spinal meningioma | MR-Egger | -0.765 | 1.493 | 0.610 | 0.002 | 0.023 | 0.917 | 84.029 | 69 | 0.105 | 84.042 | 70 | 0.121 |
| Hemoglobin A1c | Spinal meningioma | Maximum likelihood | -0.641 | 0.705 | 0.364 | 0.002 | 0.023 | 0.917 | 84.029 | 69 | 0.105 | 84.042 | 70 | 0.121 |
| Hemoglobin A1c | Spinal meningioma | Weighted median | -0.982 | 1.086 | 0.366 | 0.002 | 0.023 | 0.917 | 84.029 | 69 | 0.105 | 84.042 | 70 | 0.121 |
| FG | Intracranial meningioma | Inverse variance weighted | 0.022 | 0.221 | 0.919 | 0.015 | 0.012 | 0.209 | 27.411 | 36 | 0.848 | 29.045 | 37 | 0.822 |
| FG | Intracranial meningioma | MR-Egger | -0.410 | 0.404 | 0.317 | 0.015 | 0.012 | 0.209 | 27.411 | 36 | 0.848 | 29.045 | 37 | 0.822 |
| FG | Intracranial meningioma | Maximum likelihood | 0.023 | 0.222 | 0.918 | 0.015 | 0.012 | 0.209 | 27.411 | 36 | 0.848 | 29.045 | 37 | 0.822 |
| FG | Intracranial meningioma | Weighted median | 0.076 | 0.317 | 0.811 | 0.015 | 0.012 | 0.209 | 27.411 | 36 | 0.848 | 29.045 | 37 | 0.822 |
| FG | Spinal meningioma | Inverse variance weighted | 0.045 | 0.512 | 0.930 | 0.013 | 0.027 | 0.642 | 25.428 | 36 | 0.905 | 25.648 | 37 | 0.920 |
| FG | Spinal meningioma | MR-Egger | -0.322 | 0.934 | 0.732 | 0.013 | 0.027 | 0.642 | 25.428 | 36 | 0.905 | 25.648 | 37 | 0.920 |
| FG | Spinal meningioma | Maximum likelihood | 0.045 | 0.513 | 0.930 | 0.013 | 0.027 | 0.642 | 25.428 | 36 | 0.905 | 25.648 | 37 | 0.920 |
| FG | Spinal meningioma | Weighted median | -0.465 | 0.722 | 0.520 | 0.013 | 0.027 | 0.642 | 25.428 | 36 | 0.905 | 25.648 | 37 | 0.920 |
| 2hGlu | Intracranial meningioma | Inverse variance weighted | 0.009 | 0.157 | 0.955 | -0.010 | 0.040 | 0.811 | 12.616 | 11 | 0.319 | 12.685 | 12 | 0.392 |
| 2hGlu | Intracranial meningioma | MR Egger | 0.138 | 0.553 | 0.807 | -0.010 | 0.040 | 0.811 | 12.616 | 11 | 0.319 | 12.685 | 12 | 0.392 |
| 2hGlu | Intracranial meningioma | Maximum likelihood | 0.009 | 0.155 | 0.952 | -0.010 | 0.040 | 0.811 | 12.616 | 11 | 0.319 | 12.685 | 12 | 0.392 |
| 2hGlu | Intracranial meningioma | Weighted median | -0.033 | 0.212 | 0.875 | -0.010 | 0.040 | 0.811 | 12.616 | 11 | 0.319 | 12.685 | 12 | 0.392 |
| 2hGlu | Spinal meningioma | Inverse variance weighted | -0.208 | 0.453 | 0.646 | 0.083 | 0.112 | 0.475 | 18.781 | 11 | 0.065 | 19.715 | 12 | 0.073 |
| 2hGlu | Spinal meningioma | MR-Egger | -1.304 | 1.552 | 0.419 | 0.083 | 0.112 | 0.475 | 18.781 | 11 | 0.065 | 19.715 | 12 | 0.073 |
| 2hGlu | Spinal meningioma | Maximum likelihood | -0.203 | 0.359 | 0.573 | 0.083 | 0.112 | 0.475 | 18.781 | 11 | 0.065 | 19.715 | 12 | 0.073 |
| 2hGlu | Spinal meningioma | Weighted median | 0.274 | 0.516 | 0.596 | 0.083 | 0.112 | 0.475 | 18.781 | 11 | 0.065 | 19.715 | 12 | 0.073 |
| Intracranial meningioma | Type 1 diabetes | Inverse variance weighted | 0.027 | 0.045 | 0.547 | -0.006 | 0.046 | 0.910 | 0.000 | 1 | 0.985 | 0.021 | 2 | 0.990 |
| Intracranial meningioma | Type 1 diabetes | MR-Egger | 0.046 | 0.140 | 0.797 | -0.006 | 0.046 | 0.910 | 0.000 | 1 | 0.985 | 0.021 | 2 | 0.990 |
| Intracranial meningioma | Type 1 diabetes | Maximum likelihood | 0.027 | 0.045 | 0.547 | -0.006 | 0.046 | 0.910 | 0.000 | 1 | 0.985 | 0.021 | 2 | 0.990 |
| Intracranial meningioma | Type 1 diabetes | Weighted median | 0.030 | 0.053 | 0.573 | -0.006 | 0.046 | 0.910 | 0.000 | 1 | 0.985 | 0.021 | 2 | 0.990 |
| Intracranial meningioma | Type 2 diabetes | Inverse variance weighted | 0.001 | 0.001 | 0.241 | 0.000 | 0.001 | 0.778 | 0.219 | 1 | 0.640 | 0.350 | 2 | 0.839 |
| Intracranial meningioma | Type 2 diabetes | MR Egger | 0.000 | 0.003 | 0.969 | 0.000 | 0.001 | 0.778 | 0.219 | 1 | 0.640 | 0.350 | 2 | 0.839 |
| Intracranial meningioma | Type 2 diabetes | Maximum likelihood | 0.001 | 0.001 | 0.244 | 0.000 | 0.001 | 0.778 | 0.219 | 1 | 0.640 | 0.350 | 2 | 0.839 |
| Intracranial meningioma | Type 2 diabetes | Weighted median | 0.001 | 0.001 | 0.297 | 0.000 | 0.001 | 0.778 | 0.219 | 1 | 0.640 | 0.350 | 2 | 0.839 |
| Intracranial meningioma | Hemoglobin A1c | Inverse variance weighted | -0.010 | 0.010 | 0.346 | 0.013 | 0.004 | 0.182 | 0.671 | 1 | 0.413 | 12.212 | 2 | 0.002 |
| Intracranial meningioma | Hemoglobin A1c | MR Egger | -0.049 | 0.012 | 0.157 | 0.013 | 0.004 | 0.182 | 0.671 | 1 | 0.413 | 12.212 | 2 | 0.002 |
| Intracranial meningioma | Hemoglobin A1c | Maximum likelihood | -0.011 | 0.004 | 0.016 | 0.013 | 0.004 | 0.182 | 0.671 | 1 | 0.413 | 12.212 | 2 | 0.002 |
| Intracranial meningioma | Hemoglobin A1c | Weighted median | -0.008 | 0.007 | 0.248 | 0.013 | 0.004 | 0.182 | 0.671 | 1 | 0.413 | 12.212 | 2 | 0.002 |
| Intracranial meningioma | FG | Wald ratio | -0.004 | 0.009 | 0.649 | NA | NA | NA | NA | NA | NA | NA | NA | NA |
| Intracranial meningioma | 2hGlu | Wald ratio | -0.057 | 0.041 | 0.169 | NA | NA | NA | NA | NA | NA | NA | NA | NA |

**Supplementary Table S4.** MR estimates and sensitivity analyses for the causal associations between diabetes-related traits and meningioma, with Benjamini–Hochberg FDR-adjusted P values.

| **outcome** | **method** | **Beta** | **SE** | **P-value** | **OR** | **95% CI** | **egger_intercept** | **se_pleio** | **pval_pleio** | **Q_MR_Egger** | **Q_df_MR_Egger** | **Q_pval_MR_Egger** | **Q_ivw** | **Q_df_ivw** | **Q_pval_ivw** | **P-value_adjusted** |
| --- | --- | --- | --- | --- | --- | --- | --- | --- | --- | --- | --- | --- | --- | --- | --- | --- |
| Intracranial meningioma | Inverse variance weighted | 0.033 | 0.034 | 0.334 | 1.034 | 0.967, 1.105 | 0.042 | 0.016 | 0.020 | 13.281 | 16 | 0.652 | 19.907 | 17 | 0.279 | 0.955 |
| Spinal meningioma | Inverse variance weighted | -0.033 | 0.072 | 0.648 | 0.968 | 0.840, 1.114 | 0.071 | 0.038 | 0.077 | 9.885 | 16 | 0.873 | 13.452 | 17 | 0.705 | 0.955 |
| Intracranial meningioma | Inverse variance weighted | -2.594 | 1.306 | 0.047 | 0.075 | 0.006, 0.966 | 0.021 | 0.012 | 0.090 | 73.459 | 62 | 0.151 | 76.975 | 63 | 0.111 | 0.410 |
| Spinal meningioma | Inverse variance weighted | 1.067 | 2.737 | 0.697 | 2.907 | 0.014, 621.117 | 0.010 | 0.026 | 0.704 | 46.298 | 62 | 0.932 | 46.443 | 63 | 0.942 | 0.955 |
| Intracranial meningioma | Inverse variance weighted | -0.625 | 0.359 | 0.082 | 0.535 | 0.265, 1.082 | -0.002 | 0.011 | 0.840 | 97.789 | 69 | 0.013 | 97.847 | 70 | 0.016 | 0.410 |
| Spinal meningioma | Inverse variance weighted | -0.632 | 0.768 | 0.41 | 0.532 | 0.118, 2.395 | 0.002 | 0.023 | 0.917 | 84.029 | 69 | 0.105 | 84.042 | 70 | 0.121 | 0.955 |
| Intracranial meningioma | Inverse variance weighted | 0.022 | 0.221 | 0.919 | 1.022 | 0.663, 1.576 | 0.015 | 0.012 | 0.209 | 27.411 | 36 | 0.848 | 29.045 | 37 | 0.822 | 0.955 |
| Spinal meningioma | Inverse variance weighted | 0.045 | 0.512 | 0.93 | 1.046 | 0.383, 2.853 | 0.013 | 0.027 | 0.642 | 25.428 | 36 | 0.905 | 25.648 | 37 | 0.920 | 0.955 |
| Intracranial meningioma | Inverse variance weighted | 0.009 | 0.157 | 0.955 | 1.009 | 0.742, 1.373 | -0.010 | 0.040 | 0.811 | 12.616 | 11 | 0.319 | 12.685 | 12 | 0.392 | 0.955 |
| Spinal meningioma | Inverse variance weighted | -0.208 | 0.453 | 0.646 | 0.812 | 0.334, 1.974 | 0.083 | 0.112 | 0.475 | 18.781 | 11 | 0.065 | 19.715 | 12 | 0.073 | 0.955 |
| Type 1 diabetes | Inverse variance weighted | 0.027 | 0.045 | 0.547 | 1.027 | 0.941, 1.122 | -0.006 | 0.046 | 0.910 | 0.000 | 1 | 0.985 | 0.021 | 2 | 0.990 | 0.649 |
| Type 2 diabetes | Inverse variance weighted | 0.001 | 0.001 | 0.241 | 1.001 | 0.999, 1.003 | 0.000 | 0.001 | 0.778 | 0.219 | 1 | 0.640 | 0.350 | 2 | 0.839 | 0.577 |
| Hemoglobin A1c | Inverse variance weighted | -0.01 | 0.01 | 0.346 | 0.990 | 0.971, 1.010 | 0.013 | 0.004 | 0.182 | 0.671 | 1 | 0.413 | 12.212 | 2 | 0.002 | 0.577 |
| FG | Wald ratio | -0.004 | 0.009 | 0.649 | 0.996 | 0.979, 1.014 | NA | NA | NA | NA | NA | NA | NA | NA | NA | 0.649 |
| 2hGlu | Wald ratio | -0.057 | 0.041 | 0.169 | 0.945 | 0.872, 1.024 | NA | NA | NA | NA | NA | NA | NA | NA | NA | 0.577 |
